# Modeling Oncolytic Viral Therapy, Immune Checkpoint Inhibition, and the Complex Dynamics of Innate and Adaptive Immunity in Glioblastoma Treatment

**DOI:** 10.3389/fphys.2020.00151

**Published:** 2020-03-03

**Authors:** Kathleen M. Storey, Sean E. Lawler, Trachette L. Jackson

**Affiliations:** ^1^Department of Mathematics, University of Michigan, Ann Arbor, MI, United States; ^2^Department of Neurosurgery, Brigham and Women's Hospital, Boston, MA, United States

**Keywords:** oncolytic viral therapy, mathematical modeling, glioblastoma, immune checkpoint inhibitor, combination therapy, innate and adaptive immunity

## Abstract

Oncolytic viruses are of growing interest to cancer researchers and clinicians, due to their selectivity for tumor cells over healthy cells and their immunostimulatory properties. The immune response to an oncolytic virus plays a critical role in treatment efficacy. However, uncertainty remains regarding the circumstances under which the immune system either assists in eliminating tumor cells or inhibits treatment via rapid viral clearance, leading to the cessation of the immune response. In this work, we develop an ordinary differential equation model of treatment for a lethal brain tumor, glioblastoma, using an oncolytic Herpes Simplex Virus. We use a mechanistic approach to model the interactions between distinct populations of immune cells, incorporating both innate and adaptive immune responses to oncolytic viral therapy (OVT), and including a mechanism of adaptive immune suppression via the PD-1/PD-L1 checkpoint pathway. We focus on the tradeoff between viral clearance by innate immune cells and the innate immune cell-mediated recruitment of antiviral and antitumor adaptive immune cells. Our model suggests that when a tumor is treated with OVT alone, the innate immune cells' ability to clear the virus quickly after administration has a much larger impact on the treatment outcome than the adaptive immune cells' antitumor activity. Even in a highly antigenic tumor with a strong innate immune response, the faster recruitment of antitumor adaptive immune cells is not sufficient to offset the rapid viral clearance. This motivates our subsequent incorporation of an immunotherapy that inhibits the PD-1/PD-L1 checkpoint pathway by blocking PD-1, which we combine with OVT within the model. The combination therapy is most effective for a highly antigenic tumor or for intermediate levels of innate immune localization. Extreme levels of innate immune cell activity either clear the virus too quickly or fail to activate a sufficiently strong adaptive response, yielding ineffective combination therapy of GBM. Hence, we show that the innate and adaptive immune interactions significantly influence treatment response and that combining OVT with an immune checkpoint inhibitor expands the range of immune conditions that allow for tumor size reduction or clearance.

## 1. Introduction

Oncolytic viral therapy (OVT) shows promise as a cancer treatment option that selectively targets cancer cells over healthy cells. Viral therapy is also viewed as a type of immunotherapy because the viral presence stimulates an adaptive immune response (Kaufman et al., 2015). However, after decades of development, OVT has yet to become a widely used treatment option. This is likely due to the multifaceted immune response to the virus, surrounding which uncertainty remains.

This work adds to a growing literature developing mathematical models of OVT. In Wodarz (2001), Wodarz developed a model to study the virus-specific and tumor-specific cytotoxic T lymphocyte response to OVT, and determined the viral and host conditions that produce an optimal tumor response. Wodarz and Komarova (2009) and Komarova and Wodarz (2010) focus on the role of the viral infection rate and develop a general framework to study oncolytic viral dynamics. Eftimie et al. (2011) study the phenomena of multi-stability and multi-instability that arise in interactions between an oncolytic virus and adaptive immune cells, and they conclude that the immune response is primarily responsible for multi-stability, while the virus is primarily responsible for multi-instability. Eftimie and Eftimie (2018) investigate the role that two disparate types of macrophages, M1 and M2, can play in enhancing OVT, finding that polarization toward M1 or M2 phenotype can enhance OVT through either anti-tumor immune activation or increased cytotoxic activity, and that the total number of macrophages plays a consequential role in treatment outcomes. Friedman et al. (2006) consider the effect of the immunosuppressive drug, cyclophosphamide, on glioma response to OVT, and find that it decreases the percentage of uninfected tumor cells.

Many of the papers within this body of work focus on either the innate immune response or the adaptive immune response to OVT, and we build on this work by incorporating both of these branches of the immune system and focusing on the interactions between them. The innate immune system plays two major roles in response to OVT: clearance of the virus and recruitment of adaptive immune cells (McDonald and Levy, 2019). The adaptive immune system, and in particular, the CD8^+^ T cells, target tumor-associated cognate antigens, in order to specifically target and kill tumor cells. Hence, the innate immune cells play a complex role in response to OVT, potentially clearing the virus before the infection takes hold within the tumor microenvironment, while simultaneously recruiting antitumor adaptive immune cells. We investigate the circumstances under which the innate immune system either assists or hinders viral therapy, thereby providing insight regarding the barriers to successful cancer treatment.

In particular, we study the use of an oncolytic Herpes Simplex Virus (HSV) to treat glioblastoma (GBM), the most aggressive primary malignant brain tumor, killing half of all patients within a year of diagnosis, and nearly all patients within 2 years (Alexander and Cloughesy, 2017). The standard treatment of care for GBM is surgical resection, followed by concurrent radiotherapy and chemotherapy, and subsequent cycles of adjuvant chemotherapy until the tumor recurs (Stupp et al., 2005). A major impediment to GBM treatment is the frequent development of resistance to the standard chemotherapy agent, temozolomide (Hegi et al., 2005; Zhang et al., 2012). Thus, novel therapies are frequently being developed and tested for use in conjunction with, or as an alternative to, temozolomide, to effectively treat GBM. In this work, we consider the effectiveness of OVT as an alternative treatment modality, by developing and analyzing an ordinary differential equation model of GBM response to OVT.

Our results from modeling GBM response to OVT suggest that this treatment is frequently ineffective due to the inhibition of T cell activity by the PD-1/PD-L1 immune checkpoint. PD-1 (programmed cell death-1) is a protein expressed on activated T cells, and its ligand, PD-L1, is frequently upregulated on cancer cells, on innate immune cells, and on T cells (Cheng et al., 2013; Shi et al., 2013). When PD-1 on the surface of a T cell is engaged by PD-L1 on neighboring tumor or innate immune cells, the T cell becomes dysfunctional or “exhausted” and loses the ability to kill its target cells. In recent years, monoclonal antibody therapies against PD-1 and PD-L1, known as immune checkpoint inhibitors, have been developed to target the PD-1/PD-L1 pathway (Barber et al., 2006; He et al., 2015; Speranza et al., 2018). Our initial model investigations suggest the necessity of increased T cell activity in response to OVT, so we also present a second model that combines OVT and an anti-PD-1 immunotherapy, known as nivolumab.

Complex interactions frequently arise when combining cancer therapies, so a number of mathematical models have been developed to study combination treatments. To highlight a few examples, de Pillis et al. (2006) develop a model of tumor response to a combination of chemotherapy and immunotherapy. In Lai and Friedman (2017) model the combination of a cancer vaccine that activates dendritic cells with an immune checkpoint inhibitor, finding that these treatments work effectively together, and developing a notion of synergy between the drugs. In Bagheri et al. (2011) model the combination of an oncolytic adenovirus with MEK-inhibitor treatment. Kim et al. (2018, 2019) investigate the effect of combining OVT, natural killer cell treatment, and a proteasome inhibitor known as bortezomib, suggesting dosing strategies that account for factors in the tumor microenvironment. Our work supplements the existing literature by investigating a combination of an oncolytic Herpes simplex virus with anti-PD-1 immunotherapy, while focusing on the crucial role of the innate immune cells in response to this treatment.

The outline of this paper is as follows: in section 2 we describe our mathematical model with OVT alone, followed by a modified version that incorporates the combination of OVT and an immune checkpoint inhibitor. In this section, we also describe the use of experimental murine data to calibrate the parameters used in the model. In section 3, we present our results with OVT alone, in section 3.1, suggesting a need to combine OVT with anti-PD-1 immunotherapy. We proceed in section 3.2 by discussing the increased efficacy of the combination therapy over OVT alone, and the multifaceted role of the innate immune system in response to the combination therapy. We summarize these results and discuss future directions in section 4.

## 2. Materials and Methods

We have developed a model to investigate the treatment of GBM through OVT and an immune checkpoint inhibitor. We use *in vivo* parameter values to simulate GBM in a murine model. We first present a model including OVT alone, and then we present an additional equation to incorporate the immune checkpoint inhibitor. In the initial set of seven equations, we model the temporal changes in five immune/cancer cell types; the oncolytic virus, which we assume to be HSV; and the molar concentration of PD-1 molecules expressed by the cells within the model. In the second version of the model, we modify the equation for the molar concentration of PD-1, and add an equation describing the molar concentration of an anti-PD-1 immunotherapy drug, which we assume to be nivolumab. The initial set of seven variables are listed in Table 1.

**Table 1:** Model variables.

**Variable**	**Description**	**Units**
*T*_*s*_	Susceptible tumor cells	#
*T*_*I*_	Infected tumor cells	#
*V*	Free viral particles	pfu
*Z*	Activated innate immune cells	#
*Y*_*T*_	Adaptive tumor-specific immune cells	#
*Y*_*V*_	Adaptive virus-specific immune cells	#
*P*	Concentration of PD-1	μ*M*

**Figure 1:**
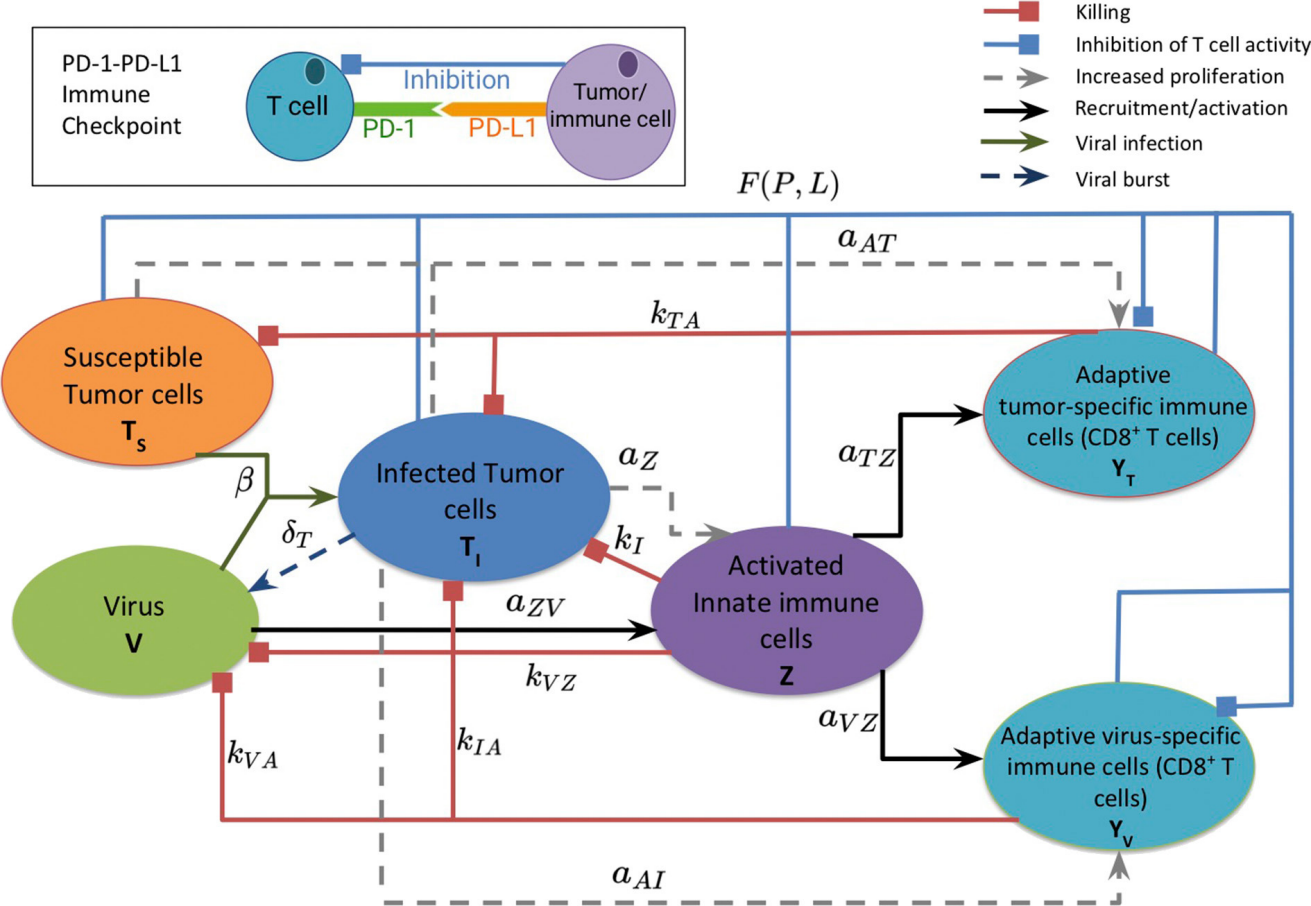
A schematic diagram of the model, depicting the interactions between the susceptible and infected tumor cells, innate and adaptive immune cells, and the virus. Susceptible tumor cells become infected by the oncolytic virus, and following successful viral replication within the infected cells, these cells lyse to give rise to new viral particles. Innate immune cells, representing both macrophages and natural killer cells, become activated when they encounter the virus or viral antigens on the infected cells, and subsequently target and kill viral particles and infected cells. The innate immune cells present viral and tumor antigens to CD8^+^ T cells, which activate antiviral and antitumor T cells. Antiviral T cells attack and kill infected cells and viral particles, and antitumor T cells target both infected and susceptible tumor cells. The inset diagram displays the inhibition of adaptive immune cell activity by the PD-1/PD-L1 immune checkpoint.

Figure 1 provides a visual representation of the model. The labeled connections between cell types in Figure 1 correspond to specific terms in the model equations described in the next section. Model parameters and their sources are listed in Table 2, and the process used to estimate them can be found in section 2.2 and in the Appendix.

**Table 2:** Model parameters.

	**Parameter**	**Description**	**Baseline**	**Range**	**References**
1	*r*_*t*_	Tumor cell growth rate	0.0192 h^−1^	0.005–0.05	Friedman et al., 2006
2	*C*_*T*_	Tumor cell carrying capacity	5.157 × 10^8^ cells	10^7^−10^9^	Fit from Linsenmann et al., 2019
3	β	Viral infection rate	2.5 × 10^−9^ pfu^−1^h^−1^	2.5 × 10^−13^ − 2.5 × 10^−7^	Okamoto et al., 2014, Est.
4	δ_*T*_	Death rate of infected tumor cells	$\frac{1}{18}$ h^−1^	1/48 − 1/9	Friedman et al., 2006
5	ω	Viral clearance rate	0.025 h^−1^	0.001–1	Friedman et al., 2006
6	*b*_*T*_	Burst size of infected cells	50 pfu/cell	10–1,350	Friedman et al., 2006
7	*a*_*Z*_	Rate of infected cell-mediated proliferation of innate immune cells	2.4 × 10^−6^ cell^−1^h^−1^	2.4 × 10^−8^ − 2.4 × 10^−4^	Estim.
8	*a*_*ZV*_	Virus-mediated activation rate of resting innate immune cells	0.1 pfu^−1^h^−1^	2.4 × 10^−4^ − 0.2	Reynolds et al., 2006
9	δ_*Z*_	Death rate of innate immune cells	0.008 h^−1^	5 × 10^−4^ − 1/12	Eftimie and Eftimie, 2018
10	δ_*YT*_	Death rate of tumor-specific adaptive immune cells	3.75 × 10^−4^ h^−1^	0.001 − 0.0074	Banerjee et al., 2015; Mahasa et al., 2017
11	δ_*YV*_	Death rate of virus-specific adaptive immune cells	5.54 × 10^−3^ h^−1^	0.001–0.01	Mahasa et al., 2017
12	*k*_*I*_	Killing rate of infected cells by innate immune cells	0.02 cell^−1^h^−1^	0.001–0.1	Estim.
13	*k*_*VZ*_	Killing rate of virions by innate immune cells	0.005 cell^−1^h^−1^	0.001 − 2	Est., Reynolds et al., 2006
14	*k*_*TA*_	Killing rate of tumor cells by tumor-specific adaptive immune cells	$\frac{1}{24}$ cell^−1^h^−1^	0.0004–0.2	Mahasa et al., 2017
15	*k*_*IA*_	Killing rate of infected cells by virus-specific adaptive immune cells	$\frac{1}{24}$ cell^−1^h^−1^	0.0004–0.2	Mahasa et al., 2017
16	*k*_*VA*_	Killing rate of virions by virus-specific adaptive immune cells	10^−5^ cell^−1^h^−1^	10^−6^ − 10^−3^	Estim.
17	*a*_*TZ*_	Activation rate of tumor-specific adaptive immune cells via innate immune cells	0.025 h^−1^	10^−3^ − 0.1	Estim.
18	*a*_*VZ*_	Activation rate of virus-specific adaptive immune cells via innate immune cells	0.025 h^−1^	10^−3^−0.1	Estim.
19	*a*_*AT*_	Rate of tumor cell-mediated proliferation of tumor-specific adaptive immune cells	0.0016 cell^−1^h^−1^	10^−5^ − 10^−1^	Mahasa et al., 2017
20	*a*_*AI*_	Rate of infected cell-mediated proliferation of virus-specific adaptive immune cells	0.025 cell^−1^h^−1^	10^−5^ − 0.1042	Mahasa et al., 2017
21	*h*_*T*_	Half-saturation constant of tumor cells	2.7 × 10^4^ cells	40–10^5^	Banerjee et al., 2015; Mahasa et al., 2017
22	*h*_*I*_	Half-saturation constant of infected tumor cells	10^4^ cells	20–5 × 10^4^	Banerjee et al., 2015, Est.

### 2.1. Model Equations

The initial model consists of a system of seven non-linear differential equations, listed below. Each equation describes the rate of change in hours of the population of a single cell type or of the virus. We assume that the innate immune cell population includes both macrophages and natural killer cells, due to the positive feedback loop that exists between these two cellular types. Macrophages engulf and destroy viral particles, while natural killer cells primarily target and kill infected cells, so in our model we assume the collective group of innate immune cells are activated by and target both viral particles and infected tumor cells. The innate immune cells release cytokines, which recruit adaptive CD8^+^ T cells, and we assume that the T cells can be divided into two groups that primarily target either viral antigens or tumor antigens (McDonald and Levy, 2019). Following Lai and Friedman (2017) and Nikolopoulou et al. (2018), we incorporate suppression of these adaptive immune cells via the PD-1/PD-L1 checkpoint with the factor *F*(*P, L*) in Equations (5),(6).

We start simulations with the initial conditions $X_{s}=10^{5}$ cells, *V* = 10^7^ pfu (plaque-forming units), and all other cell populations beginning at 0. Time *t* = 0 represents the time at which the initial viral dose is administered, and we assume that any pre-treatment antitumor immune activity is factored into the net tumor growth rate, so there are no new immune cells being recruited to target the tumor at the time of the initial viral dose.

#### 2.1.1. Oncolytic Viral Therapy Alone

**Figure 2:**
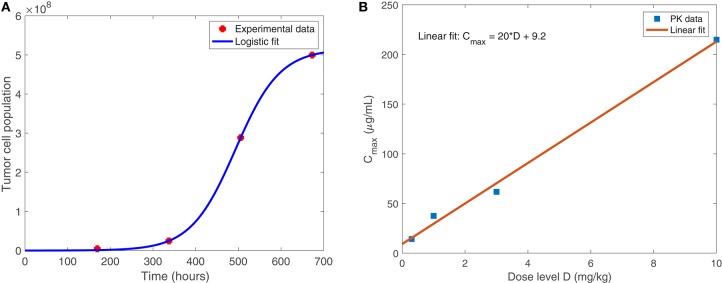
**(A)** Shows the logistic growth fit to murine GBM size data in the absence of treatment, from Linsenmann et al. (2019). We use this to determine the net growth rate, *r*_*t*_ = 0.0192 h^−1^, and carrying capacity, $C_{T}=5.157\times 10^{8}$ cells. **(B)** Displays the linear relationship between the administered dose (mg/kg) of nivolumab and the maximum plasma concentration, *C*_*max*_ (μg/mL). Data is obtained from Brahmer et al. (2010).

Equation (1), shown below, models the susceptible tumor population. The term (1a) represents logistic growth of the susceptible tumor cells with intrinsic growth rate *r*_*t*_, and with a carrying capacity *C*_*T*_ for all tumor cells. We assume a baseline growth rate of 0.0192 per hour, corresponding to a tumor doubling rate of about 35 days, and a carrying capacity of 5.157 × 10^8^ cells. We obtained these values by fitting a logistic growth curve to control data in Linsenmann et al. (2019), displayed in Figure 2A. Note that in Friedman et al. (2006), they use a similar value of 0.02 h^−1^, based on the growth of glioma cells.

The term β*T*_*s*_*V* represents viral infection of susceptible tumor cells at rate β, which shifts tumor cells from the susceptible population to the infected population, *T*_*I*_. We assume a baseline viral infection rate of 2.5 × 10^−9^ virion^−1^h^−1^, but vary this parameter in much of the numerical analysis. The final term, (1c), represents the killing of susceptible tumor cells by tumor-specific adaptive immune cells, *Y*_*T*_. We use Michaelis-Menten kinetics to model saturation in the immune response, assuming that an over-abundance of tumor cells restricts movement within the tumor architecture (Kirschner and Panetta, 1998). The *k*_*TA*_ denotes the maximum immune killing rate of tumor cells, for which we assume a baseline value of $\frac{1}{24}$ cell^−1^h^−1^ from Mahasa et al. (2017), corresponding to each T cell killing one tumor cell each day. The parameter *h*_*T*_ represents the population of *T*_*s*_ at which the immune cells lyse tumor cells at half of their maximum killing rate. We use a baseline value of $h_{T}=2.7\times 10^{4}$ cells from Banerjee et al. (2015), but we allow for a feasible range that includes much smaller values, as seen in Mahasa et al. (2017).

(1) $$\frac{dT_{s}}{dt}=\underset{\text{(1a) Tumor growth}}{\underset{︸}{r_{t}T_{s}\left(1-\frac{T_{s}+T_{I}}{C_{T}}\right)}}-\underset{\begin{array}{l} \text{(1b) Viral}\\ \text{ infection} \end{array}}{\underset{︸}{\beta T_{s}V}}-\underset{\begin{array}{l} \text{ (1c) Adaptive}\\ \text{immune killing} \end{array}}{\underset{︸}{k_{TA}Y_{T}\frac{T_{s}}{h_{T}+T_{s}}}}$$

Equation (2) models the infected tumor population, in which term (2a) denotes the addition of cells to the population *T*_*I*_ via viral infection of susceptible tumor cells at rate β. Term (2b) denotes the death of infected cells, induced by the viral infection, at rate δ_*T*_. We assume a baseline viral lysis rate of δ_*T*_ = 1/18 h^−1^ from Friedman et al. (2006). The final three terms, (2c)–(2e), denote the killing of infected cells via innate immune cells, antitumor adaptive immune cells, and antiviral adaptive immune cells, respectively. All three types of immune killing are modeled using Michaelis-Menten kinetics, analogously to the immune killing term in Equation (2). We assume the killing rate of infected tumor cells by antiviral T cells is $k_{IA}=\frac{1}{24}$ cell^−1^h^−1^ from Mahasa et al. (2017), and we estimate a killing rate of infected tumor cells by innate immune cells with *k*_*I*_ = 0.02 cell^−1^h^−1^. We estimate this value based on the assumption that T cells primarily target infected cells, while innate immune cells primarily target the virus itself, and thus the innate immune-mediated killing rate should be smaller than the adaptive immune-mediated killing rate of infected cells.

(2) $$\begin{array}{l} \frac{dT_{I}}{dt}=\underset{\begin{array}{l} \text{(2a) Viral}\\ \text{ Infection} \end{array}}{\underset{︸}{\beta T_{s}V}}\;-\;\underset{\begin{array}{l} \text{(2b) Viral}\\ \text{ lysis} \end{array}}{\underset{︸}{\delta_{T}T_{I}}}\;-\underset{\text{(2c) Innate killing}}{\underset{︸}{k_{I}T_{I}\frac{Z}{h_{I}+Z}}}\;-\;\underset{\begin{array}{l} \text{(2d) Antitumor}\\ \text{ adaptive killing} \end{array}}{\underset{︸}{k_{TA}Y_{T}\frac{T_{I}}{h_{I}+T_{I}}}}\\ \;-\underset{\begin{array}{l} \text{ (2e) Antiviral}\\ \text{adaptive killing} \end{array}}{\underset{︸}{\;k_{IA}Y_{V}\frac{T_{I}}{h_{I}+T_{I}}}} \end{array}$$

Equation (3) models the virus population, with term (3a) representing the addition of new viral particles that are released when an infected tumor cell lyses. The parameter *b*_*T*_ denotes the viral burst size released from each infected cell, which we assume to be 50 viral particles per cell, the estimated burst size for HSV, from Friedman et al. (2006). The term *k*_*VZ*_*VZ* represents consumption of the virus by innate immune cells at rate *k*_*VZ*_. We estimate a baseline value for *k*_*VZ*_ and use the values for the rate at which primed innate immune cells consume a pathogen from Reynolds et al. (2006), and the mean rate of phagocytosis by macrophages in the presence of an unlimited supply of targets, from Branwood et al. (1992), to dictate a feasible range for *k*_*VZ*_. The term (3c) represents viral clearance by antiviral adaptive immune cells. We estimate that the adaptive immune-mediated killing rate of the virus *k*_*VA*_ << *k*_*IA*_, stemming from our assumption that the innate immune cells have a larger impact than adaptive immune cells on the clearance of the virus itself. The final term (3d), corresponds to clearance of the viral particles, resulting from local non-specific immune cells in the tumor region. We use clearance rate ω = 0.025 h^−1^ from Friedman et al. (2006), corresponding to a half-life of about 1.15 days.

(3) $$\frac{dV}{dt}=\underset{\begin{array}{l} \text{(3a) Viral}\\ \text{ burst} \end{array}}{\underset{︸}{b_{T}\delta_{T}T_{I}}}-\underset{\begin{array}{l} \text{(3b) Innate}\\ \text{ killing} \end{array}}{\underset{︸}{k_{VZ}VZ}}-\underset{\begin{array}{l} \text{(3c) Adaptive}\\ \text{ killing} \end{array}}{\underset{︸}{k_{VA}VY_{V}}}-\underset{\begin{array}{l} \text{(3d) Natural}\\ \text{ clearance} \end{array}}{\underset{︸}{\omega V}}$$

Equation (4) models the activated innate immune cell population, in which term (4a) represents the activation of resting innate immune cells. The parameter *s*_*ZR*_ denotes the rate at which new resting innate immune cells arrive in the tumor microenvironment. These resting cells are activated by interactions with the virus at rate *a*_*ZV*_, and previously activated innate immune cells recruit more resting innate immune cells at rate *a*_*Z*_*Z*, creating a positive feedback loop. We assume that the activation of resting innate immune cells occurs quickly, and thus use a quasi-steady state analysis for the resting innate immune population to obtain term (4a), as shown in Reynolds et al. (2006). We use baseline parameter values *s*_*ZR*_ = 0.08 cell h^−1^, *a*_*ZV*_ = 0.1 pfu^−1^ h^−1^, and *a*_*ZZ*_ = 0.01 cell^−1^ h^−1^ from Reynolds et al. (2006). Term (4b) represents increased proliferation of innate immune cells, induced by interactions with infected cells. Here we estimate *a*_*Z*_ to be 2.4 × 10^−6^ cell^−1^h^−1^, or equivalently 5.7 × 10^−5^ per infected cell per day. This accounts for macrophages and natural killer cells signaling to and recruiting each other, resulting in an innate immune cell positive feedback loop, with the assumption that activation occurs more commonly by encountering the virus itself, rather than by encountering infected cells. The term δ_*Z*_*Z* denotes natural innate immune cell death, at rate δ_*Z*_, which we assume to be δ_*Z*_ = 0.008 h^−1^ from Eftimie and Eftimie (2018).

(4) $$\frac{dZ}{dt}=\underset{\begin{array}{l} \text{(4a) Activation of resting}\\ \text{ innate immune cells} \end{array}}{\underset{︸}{\frac{s_{ZR}(a_{ZZ}Z+a_{ZV}V)}{\delta_{ZR}+a_{ZZ}Z+a_{ZV}V}}}+\underset{\begin{array}{l} \text{ (4b) Infected}\\ \text{cell-mediated}\\ \text{ proliferation} \end{array}}{\underset{︸}{a_{Z}T_{I}Z}}-\underset{\begin{array}{l} \text{ (4c)}\\ \text{Natural}\\ \text{ death} \end{array}}{\underset{︸}{\delta_{Z}Z}}$$

Equation (5) models the tumor-specific adaptive immune response. Term (5a) models the recruitment of T cells by innate immune cells, in which we assume a recruitment rate of *a*_*TZ*_ = 0.025 h^−1^. This is an *ad hoc* estimate, as this relationship has not been well-explored previously, and we rely on the parameter sensitivity analysis to consider a range of values for this parameter. Term (5b) represents the proliferation of adaptive T cells due to the presence of tumor antigens on both susceptible and infected tumor cells. We assume a baseline value for the tumor cell-mediated proliferation rate of tumor-specific adaptive immune cells, *a*_*AT*_, of 0.0016 h^−1^, converted from the rate in Mahasa et al. (2017). We again use Michaelis-Menten kinetics with half-saturation constant *h*_*T*_ to model the saturation of T cell activity, due to the restrictive tumor architecture. The factor *F*(*P, L*) factor represents suppression of T cell activation and proliferation via the PD-1/PD-L1 checkpoint. *P, L* denote the molar concentrations of PD-1 and PD-L1, respectively, expressed by cells within the model. The molar concentrations are obtained by first calculating the PD-1 expression on all T cells and the PD-L1 expression on all T cells, tumor cells, and innate immune cells, as outlined in the Appendix. As *P* and *L* increase, so does the number of PD-1/PD-L1 complexes within the tumor region. This increase corresponds to a smaller *F*(*P, L*) value, modeling the inhibition of T cell activity. Term (5d) represents natural death of antitumor T cells. We use $\delta_{YT}=3.75\times 10^{-4}$ h^−1^ from Mahasa et al. (2017), corresponding to a half-life of about 77 days.

(5) $$\begin{array}{l} \frac{dY_{T}}{dt}=\left(\underset{\begin{array}{l} \text{ (5a)}\\ \text{ Activation}\\ \text{ via innate}\\ \text{immune cells} \end{array}}{\underset{︸}{a_{TZ}Z}}+\underset{\begin{array}{l} \text{ (5b) Tumor cell-}\\ \text{mediated proliferation} \end{array}}{\underset{︸}{a_{AT}Y_{T}\frac{T_{s}+T_{I}}{h_{T}+T_{s}+T_{I}}}}\right)\underset{\begin{array}{l} \text{(5c) PD-1-}\\ \text{ PD-L1}\\ \text{suppression} \end{array}}{\underset{︸}{F\left(P,\;L\right)}}\\ \;-\underset{\begin{array}{l} \text{(5d) Natural}\\ \text{ death} \end{array}}{\underset{︸}{\delta_{YT}Y_{T}}} \end{array}$$

Equation (6) models the adaptive virus-specific immune response. Term (6a) represents the recruitment of CD8^+^ T cells by innate immune cells. We assume equal activation rates of antitumor and antiviral T cells via innate immune cells, so we use the same estimate, *a*_*VZ*_ = *a*_*TZ*_ = 0.025 h^−1^. Term (6b) represents the proliferation of virus-specific CD8^+^ T cells resulting from viral antigens expressed on infected cells, and we use infected cell-mediated proliferation rate *a*_*AI*_ = 0.025 cell^−1^h^−1^ from Mahasa et al. (2017). Similarly to Equation (5), the factor *F*(*P, L*) represents the PD-1/PD-L1-mediated inhibition of T cell activation and proliferation. The parameter δ_*YV*_ denotes the rate of natural cell death of antiviral T cells. We use $\delta_{YV}=5.54\times 10^{-3}$ h^−1^ from Mahasa et al. (2017), corresponding to a half-life of 5.2 days.

(6) $$\frac{dY_{V}}{dt}=\left(\underset{\begin{array}{l} \text{ (6a)}\\ \text{ Activation}\\ \text{ via innate}\\ \text{immune cells} \end{array}}{\underset{︸}{a_{VZ}Z}}+\underset{\begin{array}{l} \text{ (6b) Infected}\\ \text{ cell-mediated}\\ \text{ proliferation} \end{array}}{\underset{︸}{a_{AI}Y_{V}\frac{T_{I}}{h_{I}+T_{I}}}}\right)\underset{\begin{array}{l} \text{(6c) PD-1-}\\ \text{ PD-L1}\\ \text{suppression} \end{array}}{\underset{︸}{F\left(P,\;L\right)}}-\underset{\begin{array}{l} \text{(6d) Natural}\\ \text{ death} \end{array}}{\underset{︸}{\delta_{YV}Y_{V}}}$$

(7) $$\frac{dP}{dt}=\underset{\begin{array}{l} \text{(7a) PD-1 expression on}\\ \text{ adaptive immune cells } \end{array}}{\underset{︸}{\rho_{p}\left(\frac{dY_{T}}{dt}+\frac{dY_{V}}{dt}\right)}}$$

where $\frac{dY_{T}}{dt}$ and $\frac{dY_{V}}{dt}$ denote the expressions in Equations (5) and (6), *L* denotes the molar concentration of PD-L1 within the tumor microenvironment, represented by

(8) $$L=\underset{\begin{array}{l} \text{PD-L1 expression on adaptive immune cells,}\\ \text{ tumor cells, and innate immune cells} \end{array}}{\underset{︸}{\rho_{L}(Y_{T}+Y_{V}+\epsilon_{T}(T_{s}+T_{I})+\epsilon_{Z}Z)}}$$

and

(9) $$F(P,L)=\frac{1}{1+PL/K_{YQ}}$$

#### 2.1.2. With Immune Checkpoint Inhibitor

When we incorporate the immune checkpoint inhibitor within the model, the functional forms for Equations (1)–(6) remain the same. For the equation describing PD-1 concentration, we modify Equation (7) and replace with (10) below. We also add an eighth equation, representing the change in molar concentration of an anti-PD-1 immunotherapy drug, *A*, as follows:

(10) $$\frac{dP}{dt}=\underset{\begin{array}{l} \text{(8a) PD-1 expression on}\\ \text{ adaptive immune cells} \end{array}}{\underset{︸}{\frac{P}{Y_{T}+Y_{V}}\left(\frac{dY_{T}}{dt}+\frac{dY_{V}}{dt}\right)}}-\underset{\begin{array}{l} \text{(8b) Blocking}\\ \text{ by anti-PD-1} \end{array}}{\underset{︸}{\mu_{PA}PA}}$$

(11) $$\frac{dA}{dt}=\underset{\begin{array}{l} \text{ (9a)}\\ \text{ anti-PD-1}\\ \text{ dosing} \end{array}}{\underset{︸}{\text{A(t)}}}-\underset{\begin{array}{l} \text{9b) Depletion by}\\ \text{ blocking PD-1} \end{array}}{\underset{︸}{\mu_{PA}PA}}-\underset{\begin{array}{l} \text{ (9c)}\\ \text{ Natural}\\ \text{depletion} \end{array}}{\underset{︸}{\delta_{A}A,}}$$

In the presence of anti-PD-1 therapy, Equation (10) models the total number of free molecules of PD-1 within the tumor microenvironment. In term (8a), we replace ρ_*p*_ from Equation (7) with *P*/(*Y*_*T*_+*Y*_*V*_), since the mass of PD-1 changes when the drug binds to it. Hence the ratio between the mass of a PD-1 molecule and the mass of a T cell does not remain constant in the presence of the anti-PD-1 drug. Term (8b) models the binding of the drug to PD-1 at rate μ_*PA*_, thereby blocking the PD-1 from forming a complex with PD-L1.

In Equation (11), *A*(*t*) represents the source of the anti-PD-1 drug, which is derived from pharmacokinetic data in section 2.2. Term (9b) models the depletion of the drug as it binds to PD-1. Term (9c) represents the natural depletion of free drug that has not bound to PD-1. We estimate the parameter δ_*A*_, the natural decay rate of anti-PD-1, to be 0.0019 h^−1^, converted from the half-life of 15 days, published in Brahmer et al. (2010). In our parameter sensitivity analysis, we vary δ_*A*_ in the range 1.37 × 10^−3^ − 0.058 h^−1^, converted from the range in Nikolopoulou et al. (2018). To estimate the drug-mediated blocking rate of PD-1, μ_*PA*_, we use a similar argument to one used in Lai and Friedman (2017) to obtain μ_*PA*_ = 8.945L/μmol/h. See the Appendix for a full derivation of this parameter value.

### 2.2. Immune Checkpoint Parameter Estimation

The function in terms (5c) and (6c) is defined in Equation (9), with *L* given by (8). In the expression for *L*, the molar concentration of PD-L1 in the tumor microenvironment, ρ_*L*_, denotes the molar concentration of PD-L1 per T cell. In our simulations, we use $\rho_{L}=2.510\times 10^{-11}\mu \text{M}$. See Appendix for the full derivation of this parameter value. To complete the derivation of term (6c), we define *Q* to be the molar concentration of PD-1/PD-L1 complexes formed from the binding of PD-1 and PD-L1, modeled by

$$P+L\;\overset{\alpha_{PL}}{\underset{\delta_{Q}}{⇌}}\;Q,$$

where α_*PL*_, δ_*Q*_ are the association and dissociation rates of *Q*. As in Lai and Friedman (2017) and Nikolopoulou et al. (2018), we assume that the association and dissociation of *Q* are fast (Mautea et al., 2015), so applying a quasi-steady state argument, we can approximate *Q* using the equation:

$$Q=\frac{\alpha_{PL}}{\delta_{Q}}PL.$$

In Lai and Friedman (2017), they incorporate T cell inhibition via *Q* in the T cell differential equation by multiplying the activation terms by the following factor:

$$\frac{1}{1+Q/K_{TQ}}.$$

They define $K_{TQ}=\frac{1}{2}\bar{Q}=\frac{1}{2}\frac{\alpha_{PL}}{\delta_{Q}}\bar{P}\bar{L},$ where $\bar{P},\bar{L}$ denote the steady state quantities for *P, L*. Thus, we define $K_{YQ}=\frac{1}{2}\bar{P}\bar{L}$ so that we can rewrite the previous factor as

$$F(P,L)=\frac{1}{1+PL/K_{YQ}}.$$

We use $K_{YQ}=1.296\times 10^{-9}\mu {\text{M}}^{2}$, as determined by a process outlined in the Appendix.

Equation (7) models the micromolar concentration of PD-1 (in μmol/L) within the tumor microenvironment in the absence of anti-PD-1 treatment. PD-1 is expressed on T cells, so we can represent *P* by

$$P=\rho_{p}(Y_{T}+Y_{V}),$$

where *ρ*_*p*_ denotes the molar concentration of PD-1 per T cell. In our simulations, we use $\rho_{p}=1.259\times 10^{-11}\mu \text{M}$. See the Appendix for a full derivation of this parameter value. By differentiating this equation with respect to *t*, we obtain the equation shown for $\frac{dP}{dt}$.

The approved flat dosage regimen for nivolumab is 240 mg every 2 weeks. In Lee et al. (2018), they cite that the flat dosage results in similar exposure to 3 mg/kg. The typical treatment schedule consists of a single intravenous dose of 3 mg/kg nivolumab, administered for 1 h, once every 2 weeks. We use pharmacokinetic data from the Phase I study in Brahmer et al. (2010) to relate the dosage, *D*, in mg/kg to plasma concentration *C*_*max*_, in μg/mL. As shown in Figure 2B, we obtained the following linear relationship:

$$C_{max}(D)=20D+9.2.$$

We convert this to μM units using the molar mass of nivolumab, 1.436 × 10^−1^ g/μmol (Wishart et al., 2017). Hence, *Ĉ*_*max*_, the μM plasma concentration, is given by

$${\hat{C}}_{max}(D)=C_{max}(D)\times \frac{10^{-3}\frac{\text{ g*mL}}{\mu \text{g*L}}}{1.436\times 10^{-1}\text{ g/}\mu \text{mol}}$$

Thus,

$${\hat{C}}_{max}(D)=0.139D+0.064.$$

For simplicity, we use Ĉ_*max*_(3 mg/kg) = 0.481 μM as our baseline estimate for *A*(*t*) during the hour following each anti-PD-1 dose. Hence, for each time *t*_*d*_ at which anti-PD-1 is administered,

$$A(t)=\{\begin{array}{ll} 0.481 & t_{d}\leq t<t_{d}+1\\ 0 & \text{ otherwise.} \end{array}$$

## 3. Results

### 3.1. Oncolytic Viral Therapy Alone

First, we discuss our results for the model in the absence of an immune checkpoint inhibitor, given by Equations (1)–(7).

#### 3.1.1. Parameter Sensitivity Analysis

We perform a global parameter sensitivity analysis with OVT alone, to simulate a virtual experimental trial with 300 mice, each with distinct tumor and immune characteristics. We use this analysis to identify the parameters that most significantly contribute to treatment efficacy. We first determined a reasonable range of values in which to vary each model parameter using estimates in the literature when available, and otherwise estimating based on available biological information, as shown in Tables 2, 3. We performed the sensitivity analysis using Latin hypercube sampling (LHS) and partial rank correlation coefficient (PRCC) analysis (McKay et al., 1979). See the Appendix for details describing this process.

**Table 3:** Parameters used in immune checkpoints.

	**Parameter**	**Description**	**Baseline**	**Range**	**References**
					
23	*K*_*YQ*_	Inhibition of T cells by PD-1/PD-L1	1.296 × 10^−9^ (μM)^2^	10^−10^ − 10^−8^	Lai and Friedman, 2017, Est.
24	ρ_*p*_	Molar concentration of PD-1 per T cell	1.259 × 10^−11^ μM	10^−12^ − 10^−10^	Nikolopoulou et al., 2018, Est.
25	ρ_*L*_	Molar concentration of PD-L1 per T cell	2.510 × 10^−11^ μM	10^−12^ − 2 × 10^−10^	Nikolopoulou et al., 2018, Est.
26	ϵ_*T*_	Expression of PD-L1 on tumor cells vs. T cells	10	1–50	Estim.
27	ϵ_*Z*_	Expression of PD-L1 on innate immune cells vs. T cells	10	1–50	Estim.
28	*s*_*ZR*_	Source of the resting innate immune cells	0.08 cell h^−1^	0.005 − 0.2	Reynolds et al., 2006
29	*a*_*ZZ*_	Activation of resting innate immune cells by previously activated innate immune cells	0.01 cell^−1^h^−1^	0.005 − 0.2	Reynolds et al., 2006
30	δ_*ZR*_	Death rate of resting innate immune cells	0.12 cell^−1^h^−1^	0.069 − 0.12	Reynolds et al., 2006
31	δ_*A*_	Decay rate of anti-PD-1	0.0019 h^−1^	1.37 × 10^−3^ − 0.058	Brahmer et al., 2010; Nikolopoulou et al., 2018
32	μ_*PA*_	Anti-PD-1 blocking rate of PD-1	8.945 L/μ mol/h	6.45 − 2.73 × 10^2^	Lai and Friedman, 2017

**Figure 3:**
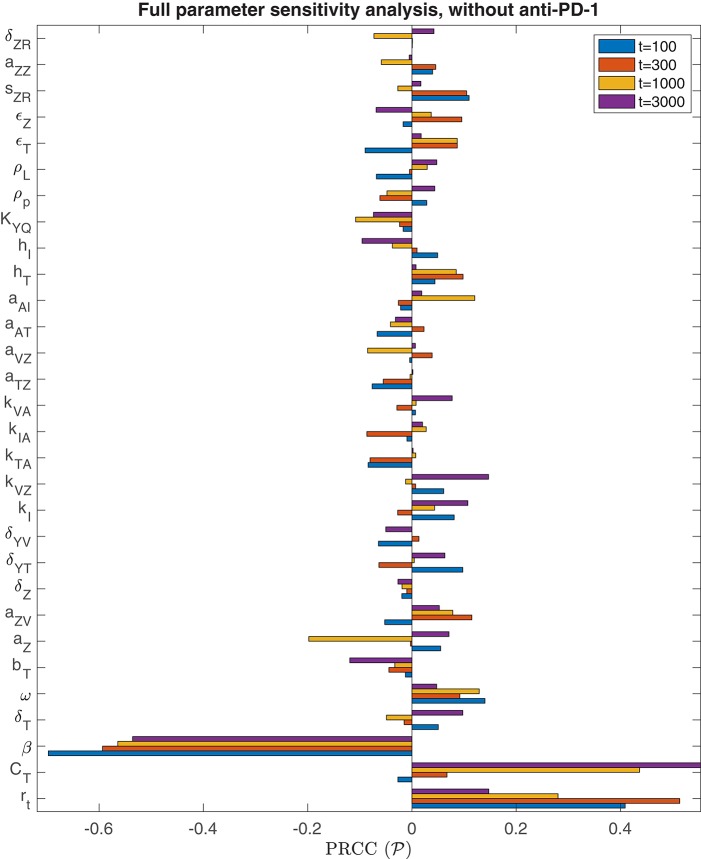
Parameter sensitivity analysis without anti-PD-1. Bar plot of the partial rank correlation coefficient between each model parameter and the susceptible tumor population at the end of the simulation, shown for simulation end points at *t* = 100, *t* = 300, *t* = 1, 000, and *t* = 3, 000 h.

We performed the global sensitivity analysis with four different simulation endpoints, at *t* = 100, *t* = 300, *t* = 1, 000, and *t* = 3, 000 h. Figure 3 depicts the PRCC for each parameter and each endpoint, determined through this global sensitivity analysis. In all cases, the parameter with the strongest relationship to the final tumor size was β, the viral infection rate. The tumor cell growth rate, *r*_*t*_, was another highly significant parameter for *t* ≤ 300. However, on the longer time scale, when *t* = 1, 000 or *t* = 3, 000, the tumor carrying capacity, *C*_*T*_, had a more significant impact on the final tumor size than the growth rate. The parameter *k*_*VZ*_, representing the innate immune-mediated killing rate of virus, also gains some significance as the simulation end time increases, but on a much smaller scale than the viral infection rate and tumor carrying capacity.

**Figure 4:**
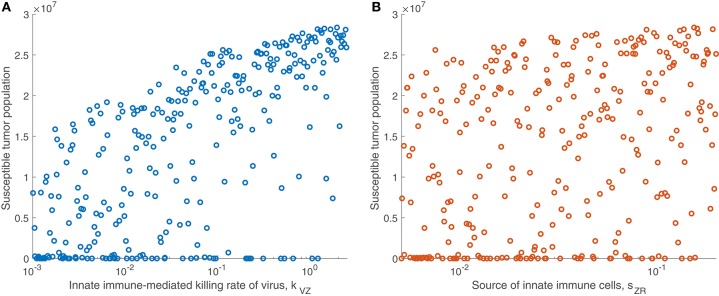
Tumor size in innate immune sensitivity analysis. The tumor size **(A)** as a function of *k*_*VZ*_, the killing rate of the virus by innate immune cells, and **(B)** as a function of *s*_*ZR*_, the source of innate immune cells. We use *t* = 300 h as the endpoint for the innate sensitivity analysis. The PRCC between *k*_*VZ*_ and the susceptible tumor population is $P\left(k_{VZ},300\right)=0.6591$, and between *s*_*ZR*_ and the susceptible tumor population is $P\left(s_{ZR},300\right)=0.3241$.

We are particularly interested in the role that the immune system plays in treatment success. In order to isolate this effect, overshadowed by the impact of the viral infection and tumor growth properties in the full sensitivity analysis, we perform another sensitivity analysis, varying only the parameters directly related to the innate immune response and fixing all other parameters. This models a trial of mice with similar tumors, treated by the same virus, but characterized by distinct innate immune responses to the treatment. We found that the most significant innate immune-related parameter on an intermediate time-scale is the innate immune-mediated killing rate of virus, *k*_*VZ*_. Using the notation P(x,t) to denote the PRCC between the parameter *x* and the tumor size after *t* h, the PRCC for *k*_*VZ*_ was $P\left(k_{VZ},300\right)=0.6591$, indicating a strong direct correlation between this parameter value and the susceptible tumor population after 300 h. The left plot in Figure 4A displays the tumor size for each simulation within the innate immune sensitivity analysis with simulation endpoint *t* = 300 h, as a function of the innate immune-mediated killing rate of virus, *k*_*VZ*_. The second most significant parameter in this analysis was the source of the innate immune cells, *s*_*ZR*_, with PRCC $P\left(s_{ZR},300\right)=0.3241$, indicating a moderate direct relationship to the post-treatment susceptible tumor population, shown in Figure 4B. The PRCC between each remaining innate immune-related parameters and the susceptible tumor population was under 0.09.

**Figure 5:**
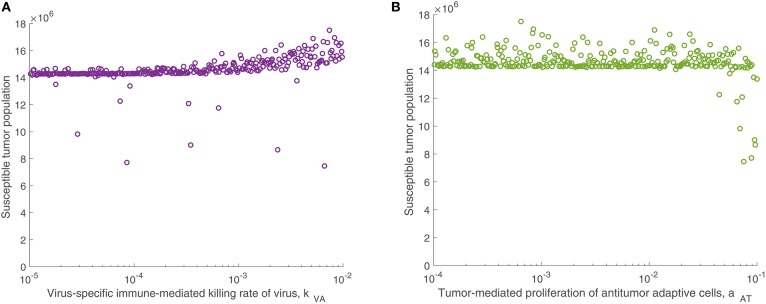
Tumor size in adaptive immune sensitivity analysis. The tumor size is shown **(A)** as a function of *k*_*VA*_, the killing rate of the virus by virus-specific adaptive immune cells, and **(B)** as a function of *a*_*AT*_, the tumor-mediated proliferation of tumor-specific adaptive immune cells. We use *t* = 300 h as the endpoint for the adaptive sensitivity analysis. The partial rank correlation coefficient (PRCC) between *k*_*VA*_ and the susceptible tumor population is $P\left(k_{VA},300\right)=0.7961$, and between *a*_*AT*_ and the susceptible tumor population is $P\left(a_{AT},300\right)=-0.1228$.

We continued this investigation by isolating the parameters directly related to the adaptive immune response and fixing all other parameters, simulating an experimental trial of mice with similar tumors and viral treatment, but characterized by distinct adaptive immune responses to the treatment. When varying only parameters related to the adaptive immune response, there was very little variation in tumor size after 300 h for most parameter sets. However, the parameter with the strongest correlation to tumor size was the virus-specific adaptive immune-mediated killing rate of virus, *k*_*VA*_, with a strong direct relationship, indicated by $P\left(k_{VA},300\right)=0.7961$. Figure 5A displays the susceptible tumor population as *k*_*VA*_ varies, and we observe that most simulations ended at a comparable high tumor size level, but for very large values of *k*_*VA*_, this post-treatment tumor size increases further, due to rapid killing of the virus by the adaptive immune cells. The second most significant parameter is the innate immune-mediated activation rate of virus-specific adaptive immune cells, *a*_*VZ*_, with $P\left(a_{VZ},300\right)=0.3128$, and the third most significant parameter is the rate of tumor cell-mediated proliferation of tumor-specific adaptive immune cells, *a*_*AT*_, which can be interpreted as the level of antigenicity of the tumor. The PRCC between this parameter and the tumor size after 300 h is $P\left(a_{AT},300\right)=-0.1228$, indicating an inverse relationship between antigenicity and the tumor size. Although *a*_*AT*_ does not have a strong correlation coefficient when compared to the parameter *k*_*VA*_, Figure 5B shows that the only simulations resulting in a reduced tumor size had high levels of antigenicity. Thus, there seems to be an important range of *a*_*AT*_ that allows for more successful therapy results, making the tumor antigenicity level potentially more interesting than the adaptive immune-mediated killing rate of virus.

#### 3.1.2. Treatment Dependence on Viral Infection Rate

**Figure 6:**
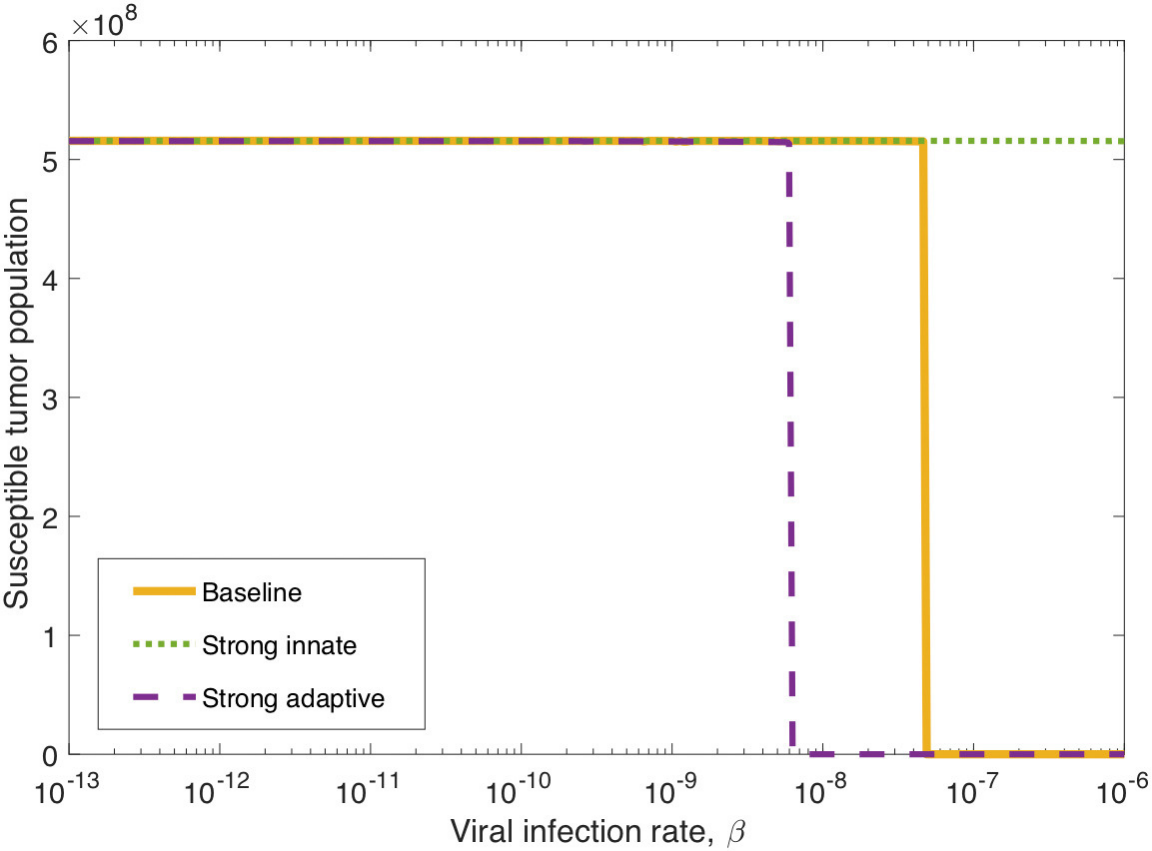
Long-term tumor size, after 3,000 h, as the viral infection rate, β varies. The tumors were treated with a single oncolytic viral dose, and we compare between a baseline tumor and a tumor with strong innate or a strong adaptive immune system. In the strong innate case, *k*_*VZ*_ = 2 and *s*_*ZR*_ = 0.2. In the strong adaptive case, *a*_*AT*_ = 0.05.

We observed in the global sensitivity analysis that the effectiveness of oncolytic viral therapy to treat GBM is highly dependent on the viral infection rate. The infectivity of an oncolytic virus is not an intrinsic property of the system; this viral characteristic can be genetically modified via gene deletions, so it is undoubtedly a parameter worthy of investigation. We investigate the effect of the viral infectivity by fixing all other parameters at their baseline level while varying only the viral infection rate, β. Due to uncertainty regarding a biologically achievable upper bound for viral infectivity, we let β vary in a large range, for the purpose of identifying the level of infectivity required for successful treatment. For each distinct β level, we simulate the model until *t* = 3, 000, when all populations have settled toward their steady state behavior. Figure 6 shows in yellow that there is a clear threshold, β ≈ 4.9 × 10^−8^, above which the tumor is eliminated through treatment, and below which the tumor reaches its carrying capacity. There is little available information about specific limitations for viable oncolytic viral infection rates, so it may be the case that many oncolytic viruses cannot feasibly reach this high level of infectivity.

We also investigate the degree to which this critical β threshold changes as the immune landscape changes. To model a tumor in a strong innate immune environment, we increase the two most influential innate immune parameters, *k*_*VZ*_ and *s*_*ZR*_, to the upper bounds of the ranges over which we vary these parameters in the sensitivity analysis, i.e., to *k*_*VZ*_ = 2 cell^−1^h^−1^ and *s*_*ZR*_ = 0.2 cell h^−1^. As β varies, the dotted green line in Figure 6 shows that the strong innate immunity prevents treatment success for all levels of viral infectivity, which we hypothesize is due to the rapid innate immune-mediated clearance of all viral particles.

It may also be the case that the oncolytic viral treatment is administered to a tumor that elicits a strong adaptive immune response. In order to test the benefit that the strong adaptive immune response may confer to treatment response, we increase the antigenicity parameter *a*_*AT*_, whose upper range yielded a reduced tumor size in the sensitivity analysis, to 0.05 cell^−1^h^−1^. As β varies, the dashed purple curve in Figure 6 shows that the viral infection threshold shifts downward from the baseline case, suggesting that in an environment with a strong adaptive immune response, treatment can be effective with a less infectious virus, due to the increase in tumor-mediated recruitment of adaptive immune cells.

#### 3.1.3. Innate Immune Suppression of OVT

**Figure 7:**
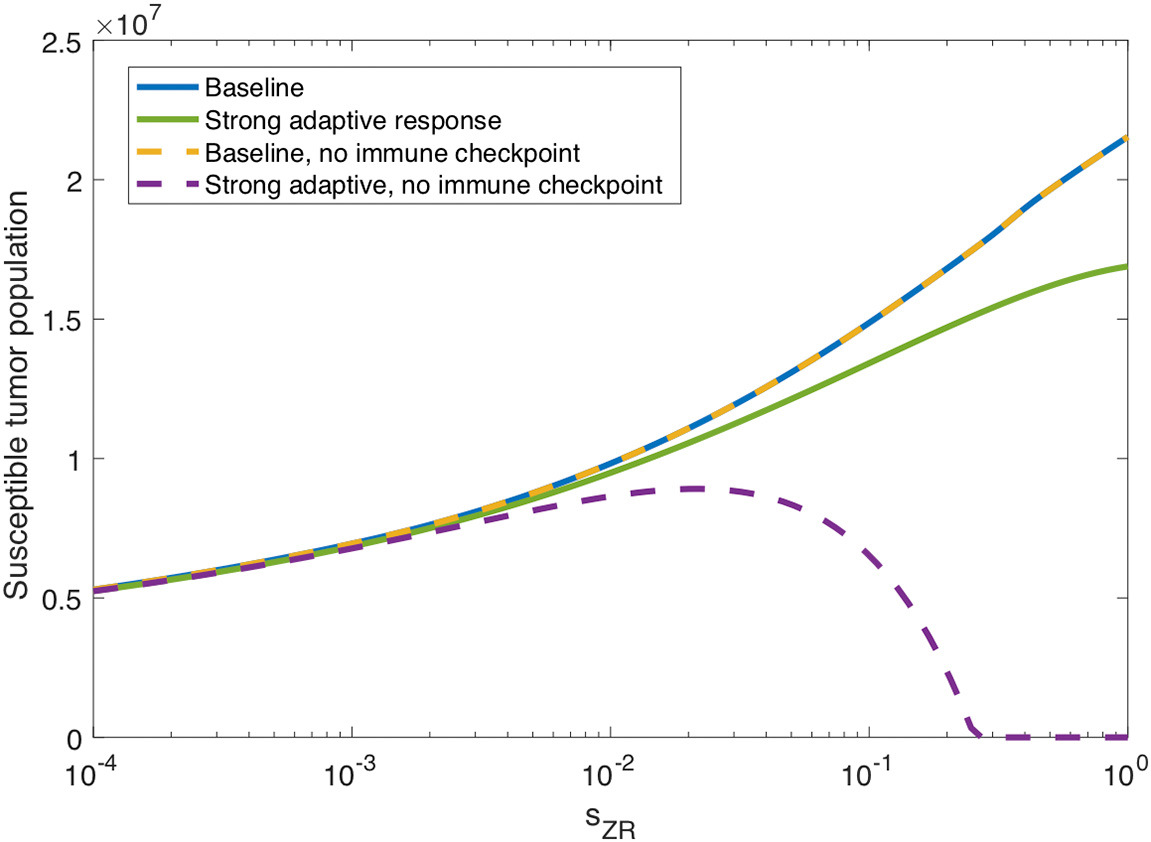
Innate immunity tradeoff. We display the susceptible tumor population after 300 h without anti-PD-1, as the source of innate immune cells, *s*_*ZR*_ varies. We compare the baseline parameter regime with a stronger adaptive immune response, given by *a*_*AT*_ = 0.05, in the presence of suppression of T-cell activity via the PD-1/PD-L1 pathway and also in the absence of T cell suppression via the PD-1/PD-L1 pathway.

In the previous subsection, we observe that on its own, a strong innate immune response negatively impacts the tumor response to OVT. However, intuition suggests that when paired with a strong adaptive immune response, for a sufficiently strong innate response, the innate immune cell recruitment of adaptive immune cells could potentially outweigh the rapid clearance of the virus. In Figure 7, we consider the tumor size after 300 h as the source of innate immune cells, *s*_*ZR*_ varies. The curve in blue shows a monotone increase in tumor size in the baseline case, as *s*_*ZR*_ increases. The green curve shows the results in a tumor microenvironment with a strong adaptive immune response, modeled as before, with a high level of tumor antigenicity, *a*_*AT*_ = 0.05 cell^−1^h^−1^. In this case, the tumor size again increases monotonically with *s*_*ZR*_, albeit deviating to some extent from the baseline case for large *s*_*ZR*_ values. The monotonic behavior suggests that even when paired with a strong adaptive immune response, the strong innate immune system is not beneficial to OVT response. Hence, with a larger innate immune presence, the faster recruitment of adaptive immune cells is not sufficient to offset the rapid viral clearance from the innate immune cells.

However, in the absence of PD-1/PD-L1 immune suppression, i.e., when *F*(*P, L*) = 1 in Equations (5), (6), we observe the opposite trend for large *s*_*ZR*_. The dashed yellow curve in Figure 7 shows that eliminating the immune checkpoints in the baseline case has essentially no effect on the treatment response, but when paired with a strong adaptive immune response, displayed in purple, the tumor size decreases for sufficiently large *s*_*ZR*_. Hence, without the PD-1/PD-L1 suppression of T-cell activity, the faster recruitment of adaptive immune cells resulting from a large innate immune presence, can yield more effective treatment results. This suggests that for tumors with strong adaptive immunity, combining OVT with immunotherapies that inhibit the PD-1/PD-L1 checkpoint may improve treatment efficacy. These observations motivated the inclusion of anti-PD-1 immunotherapy in our model. We will discuss the results from the combination therapy model in the following section.

### 3.2. Combination Therapy With Anti-PD-1

Next, we discuss our results for the model that includes both oncolytic viral therapy and the immune checkpoint inhibitor, anti-PD-1, described by Equations (1)–(6), (10), (11).

#### 3.2.1. Parameter Sensitivity Analysis

**Figure 8:**
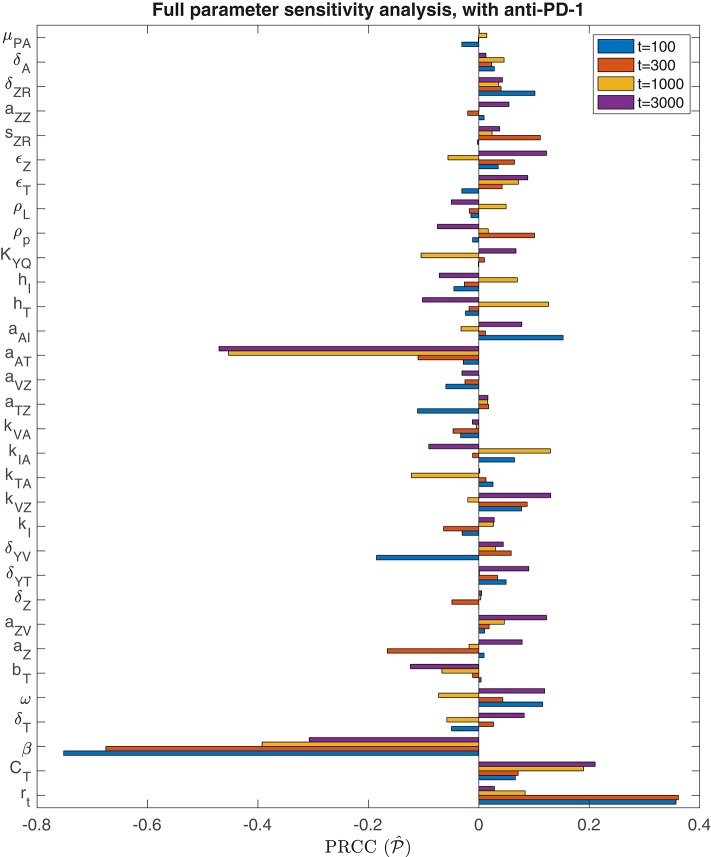
Parameter sensitivity analysis with anti-PD-1. Bar plot of PRCC values for each model parameter, shown for simulation end points at *t* = 100, *t* = 300, *t* = 1, 000, and *t* = 3, 000 h.

We also perform a parameter sensitivity analysis with both oncolytic viral therapy and anti-PD-1 immunotherapy, using the method described in section 3.1.1, in order to identify parameters that gain or lose significance with the combination therapy, when compared to the sensitivity analysis with OVT alone. Figure 8 displays the PRCC for each parameter in this global sensitivity analysis. We will represent this PRCC by $\hat{P}$ when it refers to the model with anti-PD-1. The most substantial difference between this analysis and the analysis with OVT alone relates to the parameter *a*_*AT*_, representing the level of tumor antigenicity. With anti-PD-1, the PRCC between *a*_*AT*_ and tumor size after 1,000 h is $\hat{P}\left(a_{AT},1,000\right)=-0.4532$, and after 3,000 h is $\hat{P}\left(a_{AT},3,000\right)=-0.4705$, whereas with oncolytic viral therapy alone, the corresponding PRCC values for *a*_*AT*_ are $P\left(a_{AT},1,000\right)=-0.0411$ and $P\left(a_{AT},3,000\right)=-0.0316$. Hence, the parameter *a*_*AT*_ has a much stronger correlation with post-treatment tumor size when the tumor is treated with anti-PD-1, suggesting that tumor antigenicity contributes significantly more to the effectiveness of the combination therapy than to the effectiveness of OVT alone. Otherwise, the viral infection rate, β, is still the most significant parameter for simulation end-time *t* ≤ 1, 000. For *t* = 3, 000, the parameter *a*_*AT*_ surpasses β, with $\hat{P}\left(a_{AT},3,000\right)=-0.4705$ and $\hat{P}\left(\beta ,3,000\right)=-0.3071$. We also note that the carrying capacity, *C*_*T*_, is much less significant with anti-PD-1 than with OVT alone, suggesting more effective treatment with the combination therapy, leading to more frequent tumor size reduction or clearance.

**Figure 9:**
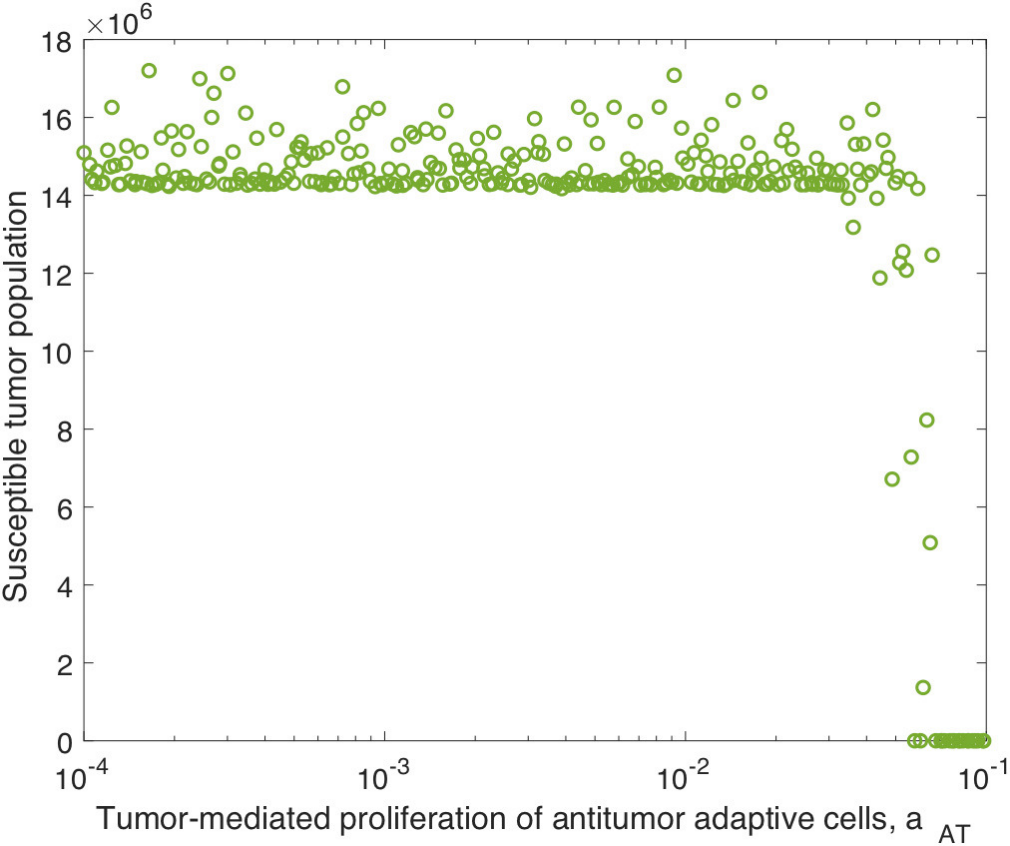
Tumor size in adaptive immune sensitivity analysis with anti-PD-1, as a function of *a*_*AT*_, the tumor-mediated proliferation of tumor-specific adaptive immune cells. We use *t* = 300 h as the endpoint for the adaptive sensitivity analysis. The PRCC between *a*_*AT*_ and the susceptible tumor population is $\hat{P}\left(a_{AT},300\right)=-0.3213$.

Analogously to the previous section, we perform additional sensitivity analyses, first varying only the parameters directly related to the innate immune response and fixing all other parameters, and subsequently varying only the parameters directly related to the adaptive immune response. In the innate immune case, the results were very similar to those with OVT alone, and we summarize these in the Appendix. Similarly to the global parameter sensitivity analysis, when we vary only adaptive immune-related parameters, the parameter *a*_*AT*_ is much more significant with anti-PD-1 than without this treatment. With anti-PD-1, the PRCC is $\hat{P}\left(a_{AT},300\right)=-0.3213$, as compared to $P\left(a_{AT},300\right)=-0.1228$ with OVT alone. This is the second most significant parameter in this analysis, surpassing the innate immune-mediated activation rate of virus-specific adaptive immune cells, *a*_*VZ*_, with $\hat{P}\left(a_{VZ},300\right)=0.2685$. The most significant parameter is again the adaptive immune-mediated viral killing rate, *k*_*VA*_, with $\hat{P}\left(k_{VA},300\right)=0.6995$, reduced from the PRCC value without anti-PD-1 of $P\left(k_{VA},300\right)=0.7961$. Although $\left|\hat{P}\left(a_{AT},300\right)\right|$ is smaller than $\left|\hat{P}\left(k_{VA},300\right)\right|$ in this adaptive immune parameter sensitivity analysis, large values of *a*_*AT*_ seem to contribute to tumor clearance, as shown in Figure 9. In contrast, the parameter *k*_*VA*_ does not seem to contribute to a reduction in tumor size, but rather, high values of *k*_*VA*_ lead to larger tumors. Hence, the tumor antigenicity level, *a*_*AT*_, seems to be the most important adaptive immune-related parameter, with respect to tumor size reduction and clearance when treated with both anti-PD-1 and OVT. Compare Figure 9 with Figure 5 to see that the tumor reduction for large *a*_*AT*_ is much more striking with anti-PD-1 than we observed without anti-PD-1.

#### 3.2.2. Treatment Dependence on Viral Infection Rate, With Anti-PD-1

**Figure 10:**
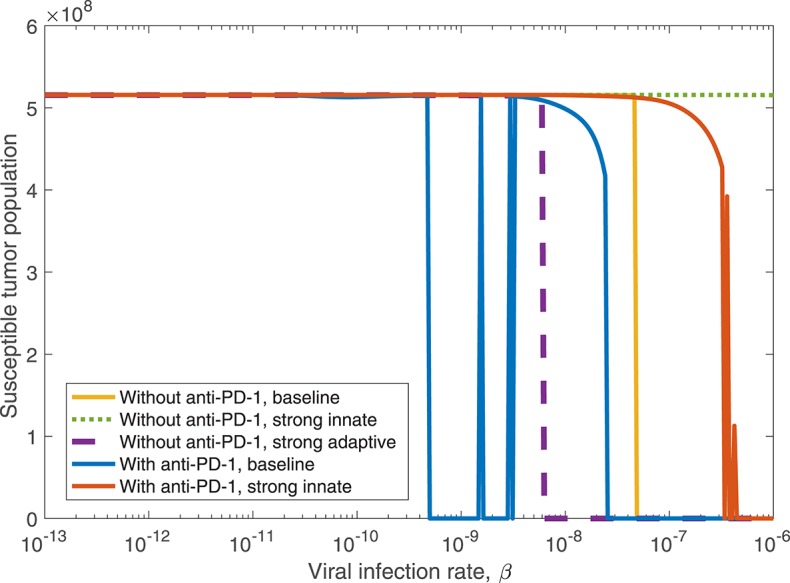
The susceptible tumor population after 3,000 h as the viral infection rate, β varies. The susceptible population is shown both with and without anti-PD-1, and we compare between a baseline tumor and a tumor with strong innate or a strong adaptive immune system. In the strong innate case, *k*_*VZ*_ = 2 and *s*_*ZR*_ = 0.2. In the strong adaptive case, *a*_*AT*_ = 0.05.

We determined in the global sensitivity analysis that the effectiveness of OVT and anti-PD-1 immunotherapy to treat GBM is dependent on the viral infection rate, but this dependence is less severe with anti-PD-1 than without. We investigate this further by varying only the viral infection rate, β, while fixing all other parameters, and comparing the tumor size after 3,000 h. In Figure 10, the blue curve displays the susceptible tumor population after treatment with anti-PD-1, with all parameters outside of β at their baseline levels. In this case, there is a larger viral infection range that will lead to tumor clearance, as compared to the yellow curve showing the tumor size after treatment with OVT alone. The threshold for tumor clearance without anti-PD-1 is β = 4.9 × 10^−8^, whereas with anti-PD-1, tumor clearance occurs for all β ≥ 2.5 × 10^−8^, and 5 × 10^−10^ < β < 3.2 × 10^−9^ will likely also lead to tumor clearance. In this range of β values, treatment success is highly sensitive to the timing of the viral infection and to the timing of the immune response. Hence, for 5 × 10^−10^ ≤ β ≤ 3.2 × 10^−9^, treatment success is likely, but the treatment results are less predictable.

**Figure 11:**
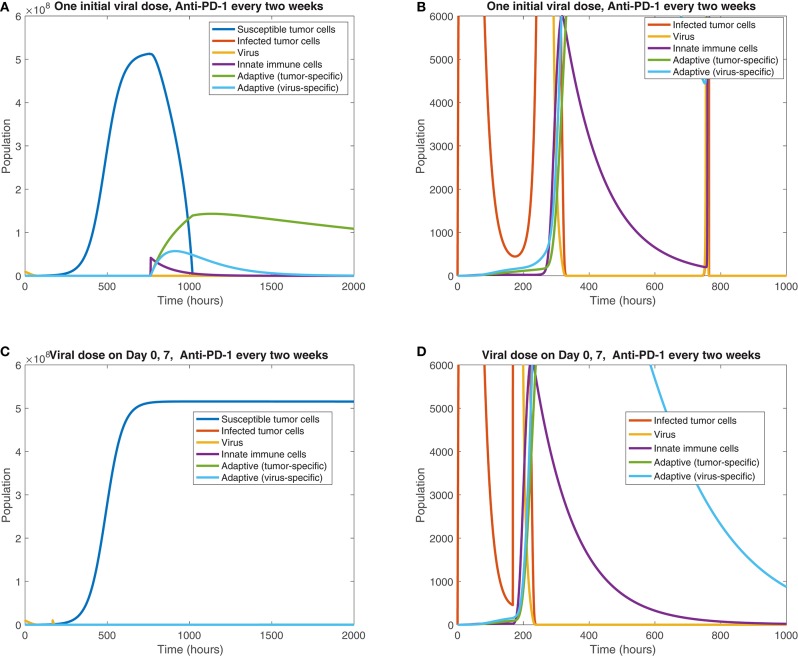
Treatment interference from a second viral dose. We display the cell populations over time in the baseline case, with anti-PD-1 dosing every 2 weeks and one initial viral dose, shown in **(A,B)**, and with one initial viral dose and a second viral dose at 1 week, shown in **(C,D)**. The second column, consisting of figures **(B,D)**, shows a zoomed in version of each of the plots to the left.

We also observe the sensitivity to infection and immune response timing when varying the dosing of the virus. Figure 11 shows the difference between the cell and viral populations when one viral dose is administered at *t* = 0, in 11(a) and 11(b), and when one initial dose is followed 7 days later by a second viral dose, in 11(c) and 11(d). In both cases, anti-PD-1 is administered intravenously for 1 h, every 2 weeks. We observe that the combination therapy results in tumor clearance when a single viral dose is administered. Interestingly, when an additional viral dose is administered 1 week after the first dose, the treatment actually becomes ineffective, with the tumor rebounding to its carrying capacity level. One possible explanation for this phenomenon is that the administration of an additional viral dose after stimulating an immune response can counteract treatment progress by diverting the attention of the immune response from the tumor alone to additional viral particles. The absence of a viral oscillation in the simulation with two doses, in comparison with the rapid viral oscillation just before 800 h in the single dose case, suggests more active immune-mediated killing of the virus when two doses are administered. It is also possible that this effect may be the result of an increased innate immune cell population, stemming from the second viral dose, which in turn produces a larger concentration of PD-L1 within the tumor microenvironment. This observation warrants follow-up work, to experimentally study the effect of multiple viral doses in combination with immune checkpoint inhibitors. The discrepancy in tumor response in these two cases emphasizes the sensitivity of the tumor response to viral and immune response timing. Additionally it suggests that the primary role of the oncolytic virus is its stimulation of the immune system, rather than its cytotoxic effect on tumor cells.

We also consider treatment dependence on viral infection rate as the immune landscape changes. In the case of a strong innate immune response, simulated using *k*_*VZ*_ = 2 and *s*_*ZR*_ = 0.2, there is a range of large β values that lead to treatment success with both anti-PD-1 and OVT, shown in red in Figure 10, in contrast to no tumor size reduction for any β with OVT alone, shown in green. However, the β range for tumor clearance with a strong innate immune response is quite high, suggesting the rapid innate immune-mediated clearance the virus prevents treatment success unless the virus is infectious enough to persist until a sufficient adaptive immune response has been initiated. Note that the results for a strong adaptive immune response, treated with both OVT and anti-PD-1 immunotherapy, are not shown in the figure because this case leads to eventual tumor clearance for all viral infection rates. Hence, for any oncolytic virus, without the PD-1/PD-L1 checkpoint suppression of adaptive immune activity, a high level of tumor-mediated adaptive immune cell proliferation is sufficient to successfully clear the tumor.

We find that in all cases, combining OVT with anti-PD-1 decreases the viral infection rate threshold for effective treatment, increasing the likelihood of developing an oncolytic virus that is sufficiently infectious to successfully treat murine GBM. However, a strong innate immune response on its own makes the therapy less effective, so we next investigate the dynamics that occur in a microenvironment equipped with both strong innate and strong adaptive immune responses.

#### 3.2.3. Innate Immunity Tradeoff, With Anti-PD-1

**Figure 12:**
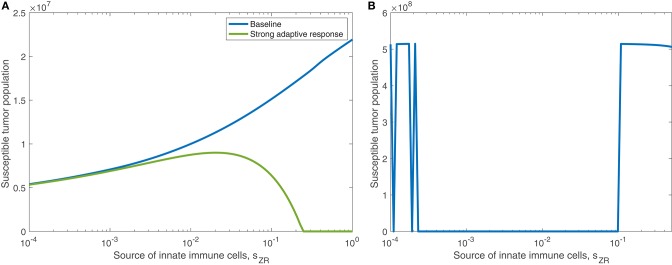
**(A)** Displays the susceptible tumor population after 300 h with anti-PD-1, as the source of innate immune cells, *s*_*ZR*_ varies. We compare the baseline parameter regime with a stronger adaptive immune response, given by *a*_*AT*_ = 0.05. **(B)** Displays the number of susceptible tumor cells, after *t* = 1, 000 h, as a function of the source of innate immune cells, *s*_*ZR*_.

We find in the previous sections that the source of innate immune cells, *s*_*ZR*_, is positively correlated with post-treatment tumor size, and that increasing the innate immune cell presence in the tumor microenvironment leads to an increase in the viral infection rate threshold required for effective treatment. Hence, in a typical tumor environment, the net contribution of the innate immune cells to the combination therapy success is negative, due to their role in viral clearance. In section 3.1.3, we determined that this was the case with OVT alone, even as the strength of the adaptive immune response increased. When the tumor is treated with both OVT and anti-PD-1, Figure 12A shows the tumor size after 300 h as the source of innate immune cells, *s*_*ZR*_ varies, in the baseline case and when paired with a strong adaptive immune response, represented by an increased *a*_*AT*_. This figure is analogous for combination therapy to Figure 7 for OVT alone, and we observe that for sufficiently large *s*_*ZR*_, the tumor size actually reaches a maximum and then declines as *s*_*ZR*_ increases. This behavior confirms our hypothesis from the previous model that combining OVT with anti-PD-1 treatment allows the antitumor immune response to reach its full potential; the strong innate response, combined with a strong adaptive immune response is sufficient to clear the tumor relatively quickly. Without anti-PD-1, such a parameter regime would yield a larger tumor than in the baseline case.

In Figure 12B, we consider the dynamics within the tumor microenvironment on a longer time scale, until *t* = 1, 000 h, as *s*_*ZR*_ varies. In this figure, all other parameters are set to their baseline level, and we observe that there is a large range of *s*_*ZR*_ that leads to eventual tumor clearance. There is one small blip occurring around $s_{ZR}=4\times 10^{-4}$, in which the tumor returns to carrying capacity, due to sensitivity to the timing of the immune response; For small values of *s*_*ZR*_, there are a few discontinuities in the long-term tumor size, due to sensitivity to the timing of the immune response. The tumor rebounds to its carrying capacity when the innate immune population decline is precisely timed to prevent an oscillation of the viral population, driven by the bursting of infected cells. This viral oscillation is required to stimulate a surge in adaptive immune activity that ultimately clears the tumor. Outside of this small range, tumor clearance occurs, except in the highest ranges of *s*_*ZR*_, in which we hypothesize the influx of innate immune cells clears the virus too quickly.

**Figure 13:**
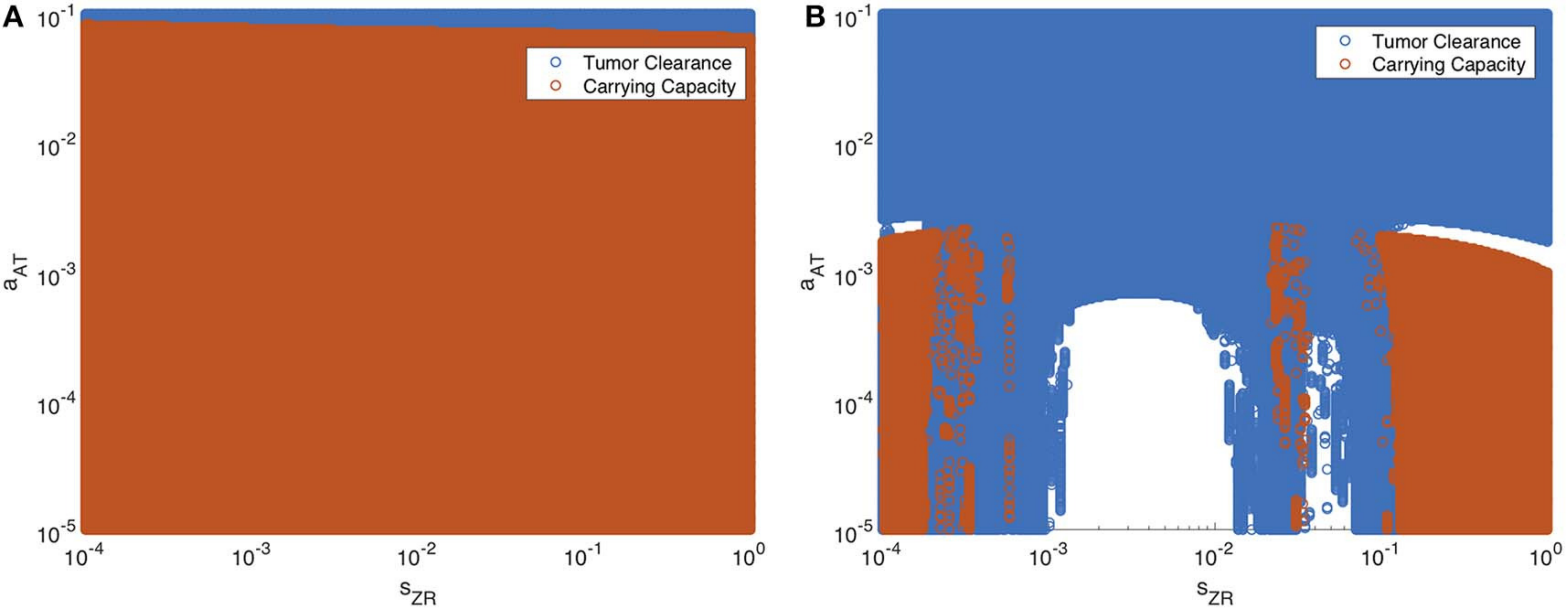
Long-term behavior as innate and adaptive immune response vary, without and with anti-PD-1. We display parameter values in the *s*_*ZR*_ − *a*_*AT*_ space that yield post-treatment tumor clearance in blue, and recurrence to tumor carrying capacity in red. The figure on the left shows the results after a single oncolytic viral dose is administered, while on the right, treatment included the initial viral dose, followed by a dose of anti-PD-1 every 2 weeks. All other parameters are set at their baseline values. **(A)** Oncolytic virus only **(B)** Oncolytic virus and anti-PD-1.

Next we vary the strength of the innate immune response and the adaptive immune response simultaneously. In Figure 13, we display the parameter values in the *s*_*ZR*_ − *a*_*AT*_ space that yield post-treatment tumor clearance or recurrence to tumor carrying capacity by *t* = 4, 000 h. Figure 13A shows the long-term results when the tumor is treated with OVT alone, while Figure 13B shows the results with both OVT and anti-PD-1 immunotherapy. With OVT alone, tumor clearance only occurs for a very small range of large *a*_*AT*_ values, i.e., when the tumor is highly antigenic. After combining OVT with anti-PD-1, tumor clearance occurs for a larger upper range of *a*_*AT*_, but for weak and intermediate values of *a*_*AT*_, there are ranges of innate immune levels leading to tumor clearance, interspersed with ranges leading to tumor growth. This suggests a much more complex relationship between the two facets of the immune system, when exposed to both therapies. For large and small *s*_*ZR*_ values, the tumor rebounds to its carrying capacity for all low-intermediate values of *a*_*AT*_, confirming that the long-term behavior described above for the baseline *a*_*AT*_ is representative of the behavior in the extreme *s*_*ZR*_ ranges as *a*_*AT*_ decreases. Similarly to the discontinuities seen in Figure 12B, for intermediate parameter values there are a few irregular instances interspersed within the blue clearance region, in which the innate immune response timing precludes an essential viral oscillation, thus leading to tumor rebound, rather than immune-mediated clearance of the tumor. Note that in the uncolored regions, namely for intermediate values of *s*_*ZR*_ and low values of *a*_*AT*_, the tumor starts to shrink early on, but then slowly rebounds when the adaptive immune populations begin to decline. At *t* = 4, 000, the susceptible population falls between 4 × 10^8^ and 5 × 10^8^ for all simulations in this range, illustrated by a representative simulation in Figure S3 in the Supplementary Material, and eventually by about 10^5^ h, the susceptible population falls within 0.1% of the carrying capacity.

Overall, we see that there is a significantly larger range of *a*_*AT*_ that makes the combination therapy effective, as compared to OVT alone. Additionally, there are ranges of innate immune strength that can be beneficial to the combination therapy, yielding eventual tumor clearance, which we did not see in the absence of anti-PD-1. The precise relationship between the innate and adaptive immune response to OVT and anti-PD-1 immunotherapy is still not well-understood, but our work suggests there are parameters regimes in which these operate in synergy, when the anti-PD-1 allows the antitumor adaptive immune cells to be sufficiently active.

## 4. Discussion

In this work, we first developed a model of GBM response to OVT and the resulting response from innate and adaptive immune cells. We parameterized the model using *in vivo* data from murine GBM models and performed sensitivity analyses to determine which parameters most significantly impact the tumor response to treatment. In Friedman et al. (2006), they concluded that a tumor cannot be eradicated by OVT unless the burst size is large. We found a similar limiting threshold, but in our model, this is a viral infection rate threshold, rather than burst size, below which tumor eradication is not possible. The infection rate is a modifiable viral feature, but effective oncolytic viral treatment requires an infectivity level that may not be biologically achievable. With a viral infection rate on the order of 10^−9^ pfu^−1^h^−1^, varying the strength of the adaptive immune response does not significantly improve tumor response to OVT alone, but it does increase the viral infection range under which the tumor can be eliminated. We found that a stronger innate immune response, driven primarily by an increase in the localization of the innate immune cells and the innate immune-mediated viral killing rate, leads to a less effective treatment, due to more rapid viral clearance by macrophages and natural killer cells. Even when combined with a strong adaptive immune response, the innate immune response has an antagonistic effect on OVT efficacy. Our results suggest this is due to the limitations on T cell productivity, imposed by the PD-1/PD-L1 immune checkpoints.

Thus, we chose to incorporate a second cancer treatment within the model, via an immune checkpoint inhibitor, in order to investigate the effect of this immunotherapy in combination with OVT. In this case, the viral infection rate is still the most significant parameter on the short-to-intermediate time frame. However, the tumor antigenicity level is much more significant when the tumor is treated with the combination therapy than with OVT alone. This is indicative of the fact that the adaptive immune system plays a much more significant role in response to the combination therapy than to OVT alone. Under the combination therapy, there is a larger viral infectivity range under which the tumor can be eliminated, increasing the possibility of developing a sufficiently infectious virus to combine with anti-PD-1 to eliminate murine GBM. However, there is a high degree of sensitivity to the timing of viral infection and immune response, suggesting that subsequent doses following an initial viral dose may interfere with the stimulated immune response.

In addition, there is a much more complex relationship between innate and adaptive immune cells in the presence of both OVT and anti-PD-1; under some circumstances, when treating a highly antigenic tumor, increasing the strength of the innate immune response can improve treatment efficacy. Hence, on its own, OVT is unlikely to effectively treat GBM, but combining with anti-PD-1 can lead to successful treatment, particularly when treating highly antigenic tumors. In such cases, a more rapid innate immune response enhances, rather than counteracts, the treatment. Our work builds upon Wodarz' investigations into oncolytic viral and adaptive immune interactions in Wodarz (2001), by determining innate immune conditions required for effective viral treatment.

We supplement the study in Eftimie and Eftimie (2018), which focused on the role of macrophages in response to OVT, by combining this focus with the interactions between innate and adaptive immune cells. With the inclusion of immune checkpoints within our model, our results suggest that outside of very extreme cases, tumor elimination is not possible with OVT alone. However, when combining OVT with anti-PD-1, in tumors below a certain antigenicity threshold, we confirm Eftimie's conclusion that tumor elimination strongly depends on the total number of innate immune cells. For sufficiently high levels of antigenicity, the influence of innate immune activity diminishes. In the future, we would like to include both M1 and M2 macrophages within our model framework to determine whether this distinction affects our model results. Our model suggests that it may be beneficial to perform testing of immune cell levels within the tumor microenvironment and of tumor antigenicity, in order to improve predictions of treatment efficacy. Additionally, vaccinating the host with tumor-specific antigen could help to enhance the antitumor adaptive immune response, thereby improving treatment outcomes.

A limitation of this model is that it is not spatially explicit, so it does not account for the spatial distribution of various cell types and the diffusion of the virus and anti-PD-1 drug. We plan to extend this work by incorporating spatial heterogeneity within the tumor, in order to investigate the degree to which this heterogeneity impacts treatment efficacy. Additionally we calibrate our model parameters using data from mouse models, which prevents direct translation to human patients. However, our work provides information that can be used to inform a clinical trial. We would like to follow up this work by first validating our computational predictions using experimental mouse models, administering a combination of HSV and the immunotherapy nivolumab to GBM in a range of immune landscapes. Our work also suggests that investigating the maximum level of tolerable infectivity for oncolytic viruses would benefit GBM treatment development. Subsequently, if the experiments confirm the necessary conditions and dosing protocol that yield tumor control or elimination, then this could provide the impetus for a clinical trial for GBM patients.

## Data Availability Statement

All datasets generated for this study are included in the article/Supplementary Material.

## Author Contributions

KS, SL, and TJ conceived the model and computational experiments. KS performed the computational experiments. KS and TJ analyzed the simulation model outputs. KS, SL, and TJ prepared and edited the manuscript.

### Conflict of Interest

The authors declare that the research was conducted in the absence of any commercial or financial relationships that could be construed as a potential conflict of interest.
