# Supplementary material for: Modeling Oncolytic Viral Therapy, Immune Checkpoint Inhibition, and the Complex Dynamics of Innate and Adaptive Immunity in Glioblastoma Treatment
Source: Front Physiol. 2020 Mar 3;11:151. doi: 10.3389/fphys.2020.00151 (PMC7063118; doi:10.3389/fphys.2020.00151)
Supplement: Supplementary file 1 [file Data_Sheet_1.pdf]

Modeling oncolytic viral therapy, immune checkpoint inhibition, and the complex dynamics of innate and adaptive immunity in glioblastoma treatment - Appendix

Kathleen M. Storey[1], Sean E. Lawler[2], and Trachette L. Jackson[1]

[1]*Department of Mathematics, University of Michigan*
[2]*Department of Neurosurgery, Brigham and Women's Hospital*

Model Equations

$$\frac{dT_s}{dt} = \underbrace{r_t T_s \left(1 - \frac{T_s + T_I}{C_T}\right)}_{\text{(1a) Tumor growth}} - \underbrace{\beta T_s V}_{\substack{\text{(1b) Viral}\\ \text{infection}}} - \underbrace{k_{TA} Y_T \frac{T_s}{h_T + T_s}}_{\substack{\text{(1c) Adaptive}\\ \text{immune killing}}} \tag{1}$$

$$\frac{dT_I}{dt} = \underbrace{\beta T_s V}_{\substack{\text{(2a) Viral}\\ \text{Infection}}} - \underbrace{\delta_T T_I}_{\substack{\text{(2b) Viral}\\ \text{lysis}}} - \underbrace{k_I T_I \frac{Z}{h_I + Z}}_{\text{(2c) Innate killing}} - \underbrace{k_{TA} Y_T \frac{T_I}{h_I + T_I}}_{\substack{\text{(2d) Antitumor}\\ \text{adaptive killing}}} - \underbrace{k_{IA} Y_V \frac{T_I}{h_I + T_I}}_{\substack{\text{(2e) Antiviral}\\ \text{adaptive killing}}} \tag{2}$$

$$\frac{dV}{dt} = \underbrace{b_T \delta_T T_I}_{\substack{\text{(3a) Viral}\\ \text{burst}}} - \underbrace{k_{VZ} V Z}_{\substack{\text{(3b) Innate}\\ \text{killing}}} - \underbrace{k_{VA} V Y_V}_{\substack{\text{(3c) Adaptive}\\ \text{killing}}} - \underbrace{\omega V}_{\substack{\text{(3d) Natural}\\ \text{clearance}}} \tag{3}$$

$$\frac{dZ}{dt} = \underbrace{\frac{s_{ZR}(a_{ZZ} Z + a_{ZV} V)}{\delta_{ZR} + a_{ZZ} Z + a_{ZV} V}}_{\substack{\text{(4a) Activation of resting}\\ \text{innate immune cells}}} + \underbrace{a_Z T_I Z}_{\substack{\text{(4b) Infected}\\ \text{cell-mediated}\\ \text{proliferation}}} - \underbrace{\delta_Z Z}_{\substack{\text{(4c)}\\ \text{Natural}\\ \text{death}}} \tag{4}$$

$$\frac{dY_T}{dt} = \left(\underbrace{a_{TZ} Z}_{\substack{\text{(5a)}\\ \text{Activation}\\ \text{via innate}\\ \text{immune cells}}} + \underbrace{a_{AT} Y_T \frac{T_s + T_I}{h_T + T_s + T_I}}_{\substack{\text{(5b) Tumor cell-}\\ \text{mediated proliferation}}} \right) \underbrace{F(P, L)}_{\substack{\text{(5c) PD-1-}\\ \text{PD-L1}\\ \text{suppression}}} - \underbrace{\delta_{YT} Y_T}_{\substack{\text{(5d) Natural}\\ \text{death}}} \tag{5}$$

$$\frac{dY_V}{dt} = \left(\underbrace{a_{VZ} Z}_{\substack{\text{(6a)}\\ \text{Activation}\\ \text{via innate}\\ \text{immune cells}}} + \underbrace{a_{AI} Y_V \frac{T_I}{h_I + T_I}}_{\substack{\text{(6b) Infected}\\ \text{cell-mediated}\\ \text{proliferation}}} \right) \underbrace{F(P, L)}_{\substack{\text{(6c) PD-1-}\\ \text{PD-L1}\\ \text{suppression}}} - \underbrace{\delta_{YV} Y_V}_{\substack{\text{(6d) Natural}\\ \text{death}}} \tag{6}$$

$$\frac{dP}{dt} = \underbrace{\rho_p \left(\frac{dY_T}{dt} + \frac{dY_V}{dt} \right)}_{\substack{\text{(7a) PD-1 expression on}\\ \text{adaptive immune cells}}}, \tag{7}$$

where $\frac{dY_T}{dt}$ and $\frac{dY_V}{dt}$ denote the expressions in equations (5) and (6), L denotes the molar concentration of PD-L1 within the tumor microenvironment, represented by

$$L = \underbrace{\rho_L(Y_T + Y_V + \epsilon_T(T_s + T_I) + \epsilon_Z Z)}_{\substack{\text{PD-L1 expression on adaptive immune cells,} \\ \text{tumor cells, and innate immune cells}}} \tag{8}$$

and

$$F(P, L) = \frac{1}{1 + PL/K_{YQ}} \tag{9}$$

With anti-PD-1:

$$\frac{dP}{dt} = \underbrace{\frac{P}{Y_T + Y_V}\left(\frac{dY_T}{dt} + \frac{dY_V}{dt}\right)}_{\substack{\text{(8a) PD-1 expression on} \\ \text{adaptive immune cells}}} - \underbrace{\mu_{PA} P A}_{\substack{\text{(8b) Blocking} \\ \text{by anti-PD-1}}} \tag{10}$$

$$\frac{dA}{dt} = \underbrace{A(t)}_{\substack{\text{(9a)} \\ \text{anti-PD-1} \\ \text{dosing}}} - \underbrace{\mu_{PA} P A}_{\substack{\text{(9b) Depletion by} \\ \text{blocking PD-1}}} - \underbrace{\delta_A A,}_{\substack{\text{(9c)} \\ \text{Natural} \\ \text{depletion}}} \tag{11}$$

Parameter Estimation

To determine K_{YQ}, we convert the corresponding density value of 1.365×10^{-18} g^2/cm^6 from [5] to micromolar concentration ($(\mu\text{mol/L})^2$). The molar mass of the protein PD-1 is 3.165×10^4 g/mol, and the molar mass of its ligand PD-L1 is 3.328×10^4 g/mol [4]. Thus we divide by both molar mass values to obtain:

$$K_{YQ} = 1.365 \times 10^{-12}\ \text{g}^2/\text{L}^2 \frac{1}{3.165 \times 10^4\ \text{g/mol}} \cdot \frac{1}{3.328 \times 10^4\ \text{g/mol}} \cdot 10^{12} \frac{\mu\text{mol}^2}{\text{mol}^2} = 1.296 \times 10^{-9} \mu\text{M}^2,$$

since one cm^3 is equivalent to one mL.

We estimate the volume (in cm^3) of the tumor region containing all particles in the model, as shown below:

$$\frac{5.157 \times 10^8\ \text{cells}}{(0.8\ \text{g/cm}^3)(10^9\ \text{cells/g})} = 0.6446\ \text{cm}^3, \tag{12}$$

where 5.157×10^8 cells and 0.8 g/cm^3 denote the tumor cell carrying capacity in cell number and cell density, respectively [6, 5], and a tumor weighing 1 gram is estimated to contain

10^9 cells [3].

In order to determine the value for ρ_p, we multiply the number of PD-1 proteins per T cell by the mass of a single PD-1 protein, then multiply by the molar mass of PD-1 and divide by the volume of the tumor microenvironment determined in (12). The mass of one PD-1 protein is 8.3×10^{-20} g, and the average number of PD-1 proteins expressed per T cell is 3096 [2, 8]. Note that one cm^3 is equivalent to one mL, so we obtain:

$$\rho_p = 3096 \text{ T cell}^{-1} \cdot 8.3 \times 10^{-20} \text{ g} \frac{1}{3.165 \times 10^{-2} \text{ g}/\mu\text{mol}} \frac{1}{6.446 \times 10^{-4} \text{ L}} = 1.259 \times 10^{-11} \mu\text{M}$$

Analogously for PD-L1, the mass of one PD-L1 ligand is 5.8×10^{-20} g, and the average number of PD-L1 molecules expressed per T cell is 9282 [1, 8], so we estimate ρ_L as follows:

$$\rho_L = 9282 \text{ T cell}^{-1} \cdot 5.8 \times 10^{-20} \text{ g} \frac{1}{3.328 \times 10^{-2} \text{ g}/\mu\text{mol}} \frac{1}{6.446 \times 10^{-4} \text{ L}} = 2.510 \times 10^{-11} \mu\text{M}$$

To estimate μ_{PA}, first, we assume that 10% of the anti-PD-1 drug is used in blocking PD-1 and that 90% degrades naturally. Thus,

$$\frac{\mu_{PA} PA}{0.1} = \frac{\delta_A A}{0.9},$$

so we assume that for a steady state $\bar{P}$,

$$\mu_{PA} = \delta_A / (9\bar{P}).$$

We convert the carrying capacity for PD-1 concentration of 7.47×10^{-7} g/L from [5] to molar concentration of PD-1, as follows:

$$\bar{P} = 7.47 \times 10^{-7} \text{ g/L} \frac{1}{3.165 \times 10^{-2} \text{ g}/\mu\text{mol}} = 2.36 \times 10^{-5} \mu\text{M},$$

where 3.165×10^4 g/mol is the molar mass of PD-1. Thus, we estimate the following baseline value for μ_{PA}:

$$\mu_{PA} = \frac{0.0019}{9 * 2.36 \times 10^{-5}} = 8.945 \text{ L}/\mu\text{mol/hour},$$

with a feasible range between 6.45 and 2.73×10^2 L/μmol/hour.

Parameter Sensitivity

We performed the sensitivity analysis using Latin hypercube sampling (LHS) and partial rank correlation coefficient (PRCC) analysis [7]. The LHS procedure consists of dividing

each parameter range into a specified number of subintervals and then randomly sampling from these intervals without replacement. For each parameter sampling, we simulate the model and then record the susceptible tumor population at the end of a fixed time interval. After simulating with each sampled parameter set, we calculate the partial rank correlation coefficient for each parameter in order to determine the strength of the relationship between the parameter and the tumor size. The PRCC is the regression coefficient obtained from the rank-transformed values for a given parameter and the rank-transformed values for the resulting tumor size.

Immune parameter sensitivity, with anti-PD-1

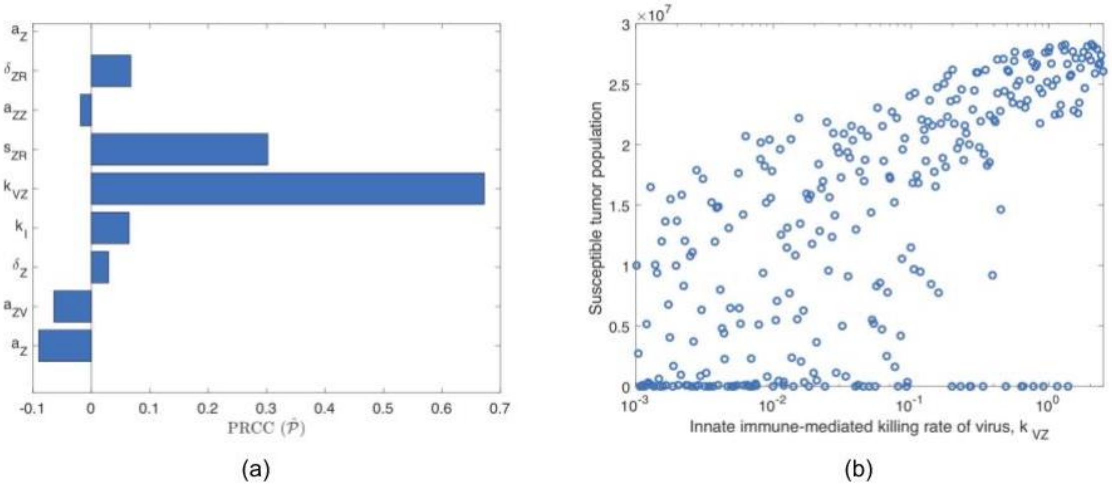

Figure 1: *Innate immune sensitivity analysis, with anti-PD-1.* In (a), we display a bar plot of PRCC values between tumor size after $t = 300$ hours and each parameter related to the innate immune response. Plot (b) shows the tumor size as a function of k_{VZ}, the killing rate of the virus by innate immune cells, in the innate immune sensitivity analysis with anti-PD-1. The partial rank correlation coefficient (PRCC) between k_{VZ} and the susceptible tumor population is $\hat{\mathcal{P}}(k_{VZ}, 300) = 0.6724$.

With the combination therapy of OVT and anti-PD-1, we performed a sensitivity analysis of only the parameters directly related to the innate immune response, while fixing all other parameters. Figure 1(a) displays a bar plot of the PRCC values between each parameter and the tumor size after 300 hours. The results of this analysis were very similar to those with OVT alone; the two most significant parameters were again the innate immune-mediated killing rate of virus, k_{VZ} and the source of the innate immune cells, s_{ZR}, with $\hat{\mathcal{P}}(k_{VZ}, 300) = 0.6724$ and $\hat{\mathcal{P}}(s_{ZR}, 300) = 0.3019$, both slightly larger than without

anti-PD-1. Figure 1(b) shows the susceptible population as a function of k_{VZ}, with anti-PD-1, displaying very similar results to the corresponding figure 4, without anti-PD-1.

As mentioned in the main text, we also performed a sensitivity analysis by varying only parameters directly related to the adaptive immune response, with the combination of OVT and anti-PD-1. Figure 2 shows the PRCC values for this analysis, between each varied parameter and the tumor size after 300 hours.

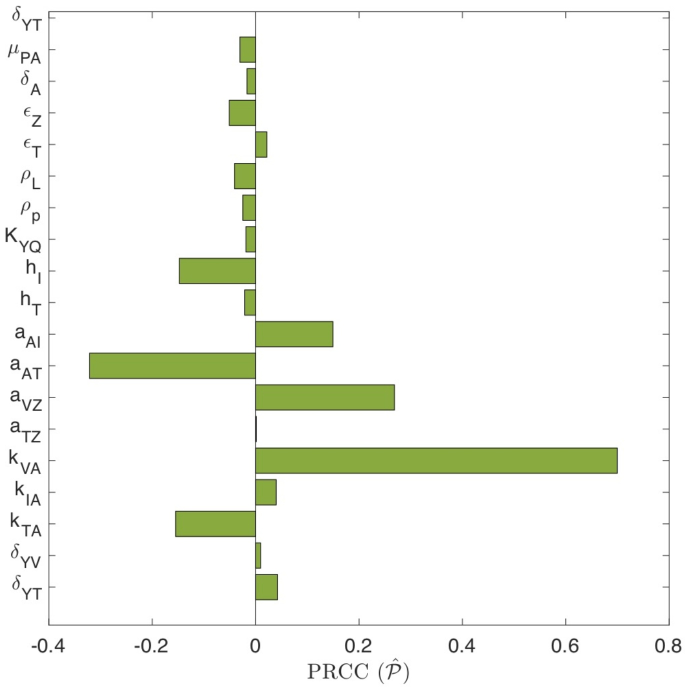

Figure 2: *Adaptive immune parameter sensitivity, with anti-PD-1.* Bar plot of PRCC values between tumor size after $t = 300$ hours and each parameter related to the adaptive immune response. In all simulations, the tumor was treated with OVT and anti-PD-1.

Additional plots

Figure 3 shows a representative simulation of the middle region from Figure 13(b), with an intermediate value for parameter s_{ZR} and low value for a_{AT}. We observe that the susceptible population begins to decrease around $t = 500$, and then once the virus has been completely eliminated, the innate immune cell population begins to decrease, causing

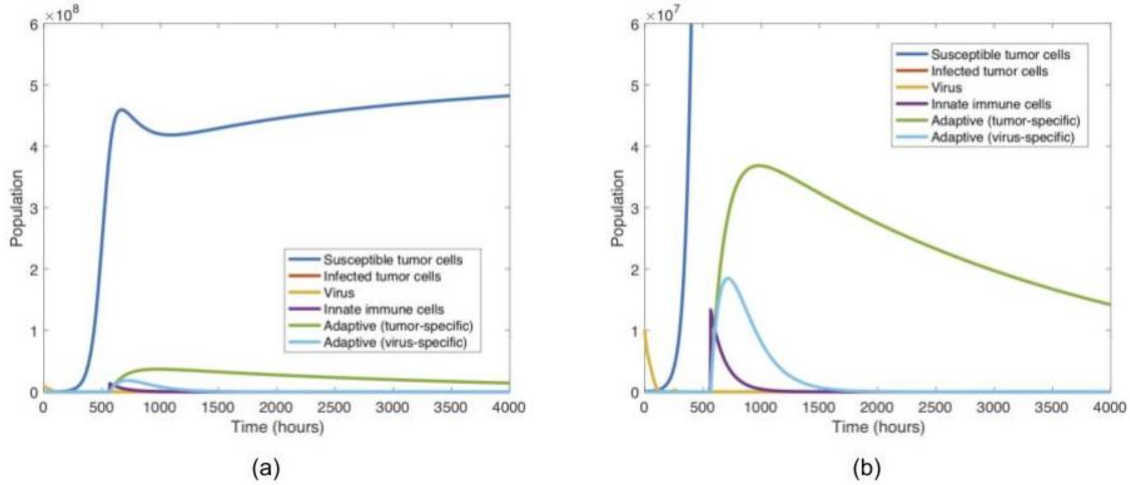

Figure 3: Cell and viral populations over time with OVT and anti-PD-1, for an intermediate innate immune source, $s_{ZR} = 0.01$ and small antigenic level, $aAT = 4.5 \times 10^{-5}$. Both plots show the same simulation, with (b) displaying a zoomed in version of plot (a).

a gradual decrease in the adaptive immune cell population. This reduction in adaptive immune cells is significant enough to allow the tumor to grow again and slowly return to its carrying capacity.

References

[1] Human PD-L1/B7-H1/CD274 protein. `http://wwwsinobiologicalcom/PD-L1-B7-H1-CD274-Protein-g-533html`.

[2] Y Agata, A Kawasaki, H Nishimura, Y Ishida, T Tsubat, H Yagita, et al. Expression of the PD-1 antigen on the surface of stimulated mouse T and B lymphocytes. Int Immunol, 8(5):765–772, 1996.

[3] U Del Monte. Does the cell number 10(9) still really fit one gram of tumor tissue? Cell Cycle, 8(3):505–506, 2009.

[4] PV Hornbeck, B Zhang, B Murray, JM Kornhauser, V Latham, and E Skrzypek. Phosphositeplus, 2014: mutations, PTMs and recalibrations. 43:D512-20, 2015.

[5] X Lai and A Friedman. Combination therapy of cancer with cancer vaccine and immune checkpoint inhibitors: A mathematical model. PLoS ONE, 12(5):e0178479, 2017.

[6] KJ Mahasa, A Eladdadi, L de Pillis, and R Ouifki. Oncolytic potency and reduced virus tumor-specificity in oncolytic virotherapy. a mathematical modelling approach. Plos One, 12(9), 2017.

[7] M. D. McKay, R. J. Beckman, and W. J. Conover. A comparison of three methods for selecting values of input variables in the analysis of output from a computer code. Technometrics, 21(2):239–245, 1979.

[8] E Nikolopoulou, LR Johnson, D Harris, JD Nagy, EC Stites, and Y Kuan. Tumour-immune dynamics with an immune checkpoint inhibitor. Letters in Biomathematics, page DOI: 10.1080/23737867.2018.1440978, 2018.
