# Supplementary figures and images for: Modeling Oncolytic Viral Therapy, Immune Checkpoint Inhibition, and the Complex Dynamics of Innate and Adaptive Immunity in Glioblastoma Treatment
Source: Front Physiol. 2020 Mar 3;11:151. doi: 10.3389/fphys.2020.00151 (PMC7063118; doi:10.3389/fphys.2020.00151)

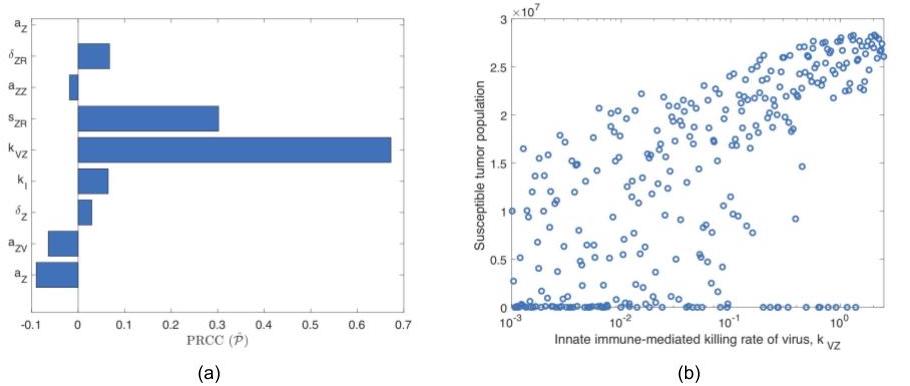

Supplement: Supplementary file 2 [file Image_1.JPEG]

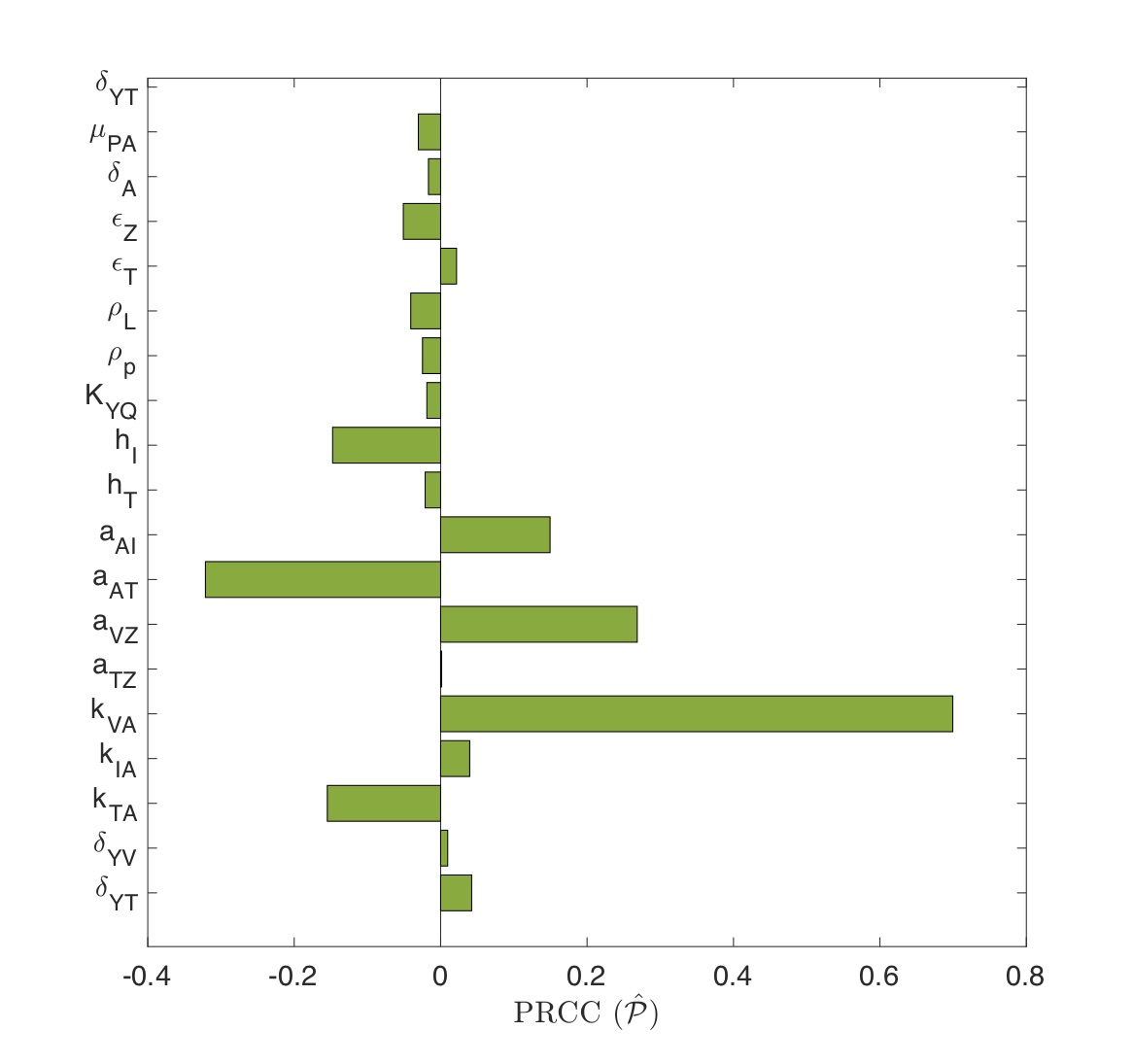

Supplement: Supplementary file 3 [file Image_2.JPEG]

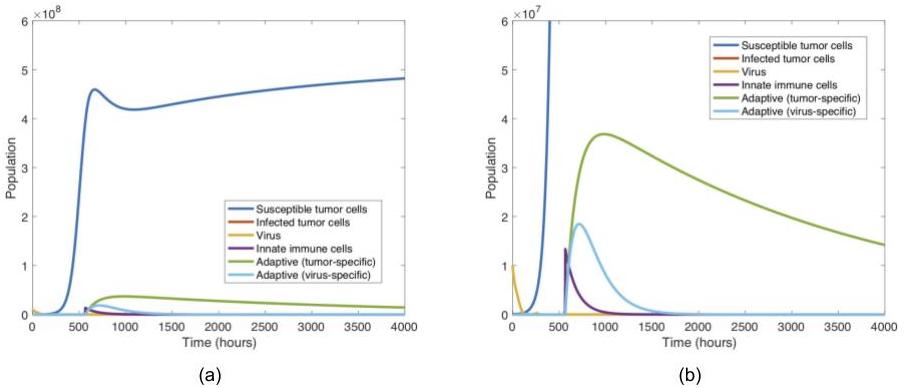

Supplement: Supplementary file 4 [file Image_3.JPEG]
